# Phytoplankton across Tropical and Subtropical Regions of the Atlantic, Indian and Pacific Oceans

**DOI:** 10.1371/journal.pone.0151699

**Published:** 2016-03-16

**Authors:** Marta Estrada, Maximino Delgado, Dolors Blasco, Mikel Latasa, Ana María Cabello, Verónica Benítez-Barrios, Eugenio Fraile-Nuez, Patricija Mozetič, Montserrat Vidal

**Affiliations:** 1 Institut de Ciències del Mar (CSIC), Barcelona, Catalunya, Spain; 2 Instituto Español de Oceanografía, Centro Oceanográfico de Gijón/Xixón, Gijón/Xixón, Asturias, Spain; 3 Instituto Español de Oceanografía, Centro Oceanográfico de Canarias, Santa Cruz de Tenerife, Tenerife, Canarias, Spain; 4 National Institute of Biology, Marine Biology Station, Piran, Slovenia; 5 Departament d’Ecologia, Universitat de Barcelona, Barcelona, Catalunya, Spain; University of Connecticut, UNITED STATES

## Abstract

We examine the large-scale distribution patterns of the nano- and microphytoplankton collected from 145 oceanic stations, at 3 m depth, the 20% light level and the depth of the subsurface chlorophyll maximum, during the Malaspina-2010 Expedition (December 2010-July 2011), which covered 15 biogeographical provinces across the Atlantic, Indian and Pacific oceans, between 35°N and 40°S. In general, the water column was stratified, the surface layers were nutrient-poor and the nano- and microplankton (hereafter phytoplankton, for simplicity, although it included also heterotrophic protists) community was dominated by dinoflagellates, other flagellates and coccolithophores, while the contribution of diatoms was only important in zones with shallow nutriclines such as the equatorial upwelling regions. We applied a principal component analysis to the correlation matrix among the abundances (after logarithmic transform) of the 76 most frequent taxa to synthesize the information contained in the phytoplankton data set. The main trends of variability identified consisted of: 1) A contrast between the community composition of the upper and the lower parts of the euphotic zone, expressed respectively by positive or negative scores of the first principal component, which was positively correlated with taxa such as the dinoflagellates *Oxytoxum minutum* and *Scrippsiella* spp., and the coccolithophores *Discosphaera tubifera* and *Syracosphaera pulchra* (HOL and HET), and negatively correlated with taxa like *Ophiaster hydroideus* (coccolithophore) and several diatoms, 2) a general abundance gradient between phytoplankton-rich regions with high abundances of dinoflagellate, coccolithophore and ciliate taxa, and phytoplankton-poor regions (second principal component), 3) differences in dominant phytoplankton and ciliate taxa among the Atlantic, the Indian and the Pacific oceans (third principal component) and 4) the occurrence of a diatom-dominated assemblage (the fourth principal component assemblage), including several pennate taxa, *Planktoniella sol*, *Hemiaulus hauckii* and *Pseudo-nitzschia* spp., in the divergence regions. Our findings indicate that consistent assemblages of co-occurring phytoplankton taxa can be identified and that their distribution is best explained by a combination in different degrees of both environmental and historical influences.

## Introduction

The oceans occupy about ¾ of the planet surface and represent the largest habitat in the biosphere. Phytoplankton, which provides about half of total primary production on Earth, supports life in this vast environment and represents a key component in the functioning of the biogeochemical cycles of the planet; therefore, understanding the response of planktonic ecosystems to hydrographical and meteorological forcing is crucial in the present context of anthropogenic global change. In particular, it is important to ascertain to what extent climate change impacts will produce alterations in the magnitude of rate processes or shifts in ecosystem structure [1]. Addressing this challenge with respect to phytoplankton, which encompasses a rich variety of taxonomic and functional groups, needs to be based on accurate descriptions of community composition. Technical developments like flow-cytometry have made a strong contribution to our knowledge of the large-scale distribution of picoplankton and the most abundant nano-sized phytoplankton organisms, and molecular techniques are contributing exciting new information on genetic diversity [2]. HPLC of photosynthetic pigments has been also a valuable tool to provide a broad view of the taxonomic composition of a phytoplankton community [3,4]. However, quantitative morpho-taxonomical information on individual taxa is still largely dependent on time-consuming microscopical observations and tends to be based on time series in long-term stations or on regional surveys. Time series provide high resolution temporal information, but have necessarily reduced spatial coverage [5–8]. On the other hand, although a number of studies have provided crucial data for some extensive marine regions like the North Sea [9], the Meridional Transects between 48°N and 50°S in the Atlantic [10] or the North Central Pacific [11], other vast areas remain relatively unexplored and global intercomparisons are hindered by different analytical and sampling procedures. Nevertheless, the current interest on whole-ocean ecosystem models makes it necessary to ascertain whether it is possible to identify distinct phytoplankton assemblages and if so, to find out how are they distributed at the relevant spatial scales. Filling this gap is crucial because many biogeochemically important functional groups, like coccolithophores, dinoflagellates and diatoms, include relatively large-sized representatives that are not well covered by methods addressing the smaller, more frequent forms. Coccolithophores are important calcifiers, dinoflagellates are motile and may use vertical migration to exploit deep nutrients in the water column and diatoms, characterized by their silica frustules, are responsible for the bulk of seasonal blooms and constitute the basis of the so-called classical food chain. In addition, according to a prevailing theory, diatoms may be responsible for a higher proportion of carbon export than could be expected from their relative abundance [12,13].

The Malaspina-2010 Expedition [14] was carried out between December 2010 and July 2011 on board R/V Hespérides and offered an exceptional opportunity to sample phytoplankton from a variety of marine areas of the world, including some poorly studied regions from the Indian and Pacific oceans. The timing of the cruise was planned so that most regions were visited during their spring- summer period, thus avoiding adverse weather and enhancing the seasonal intercomparability of the observations. Added advantages were the use of the same sampling and counting procedures for the whole data set and the fact that the same person (MD) examined all the samples, avoiding biases due to methodological differences.

This work explores the large-scale distribution patterns of nano- and microplankton as examined with the inverted microscope technique, along the seven legs of the Malaspina-2010 Expedition. For simplicity, as most taxa were photosynthetic, we will hereafter use the term “phytoplankton”, although we included ciliates and other heterotrophic forms. Basic questions addressed were: Can we define assemblages or groups of phytoplankton taxa that tend to occur together? Does the distribution of these assemblages show a consistent relationship with temperature zones or with environmental factors such as nutrient availability and water column turbulence, as proposed by Margalef [15]? Can we ascertain geographically-related differences in the composition of phytoplankton communities living under comparable ecological conditions?

## Materials and Methods

**Fig 1:**
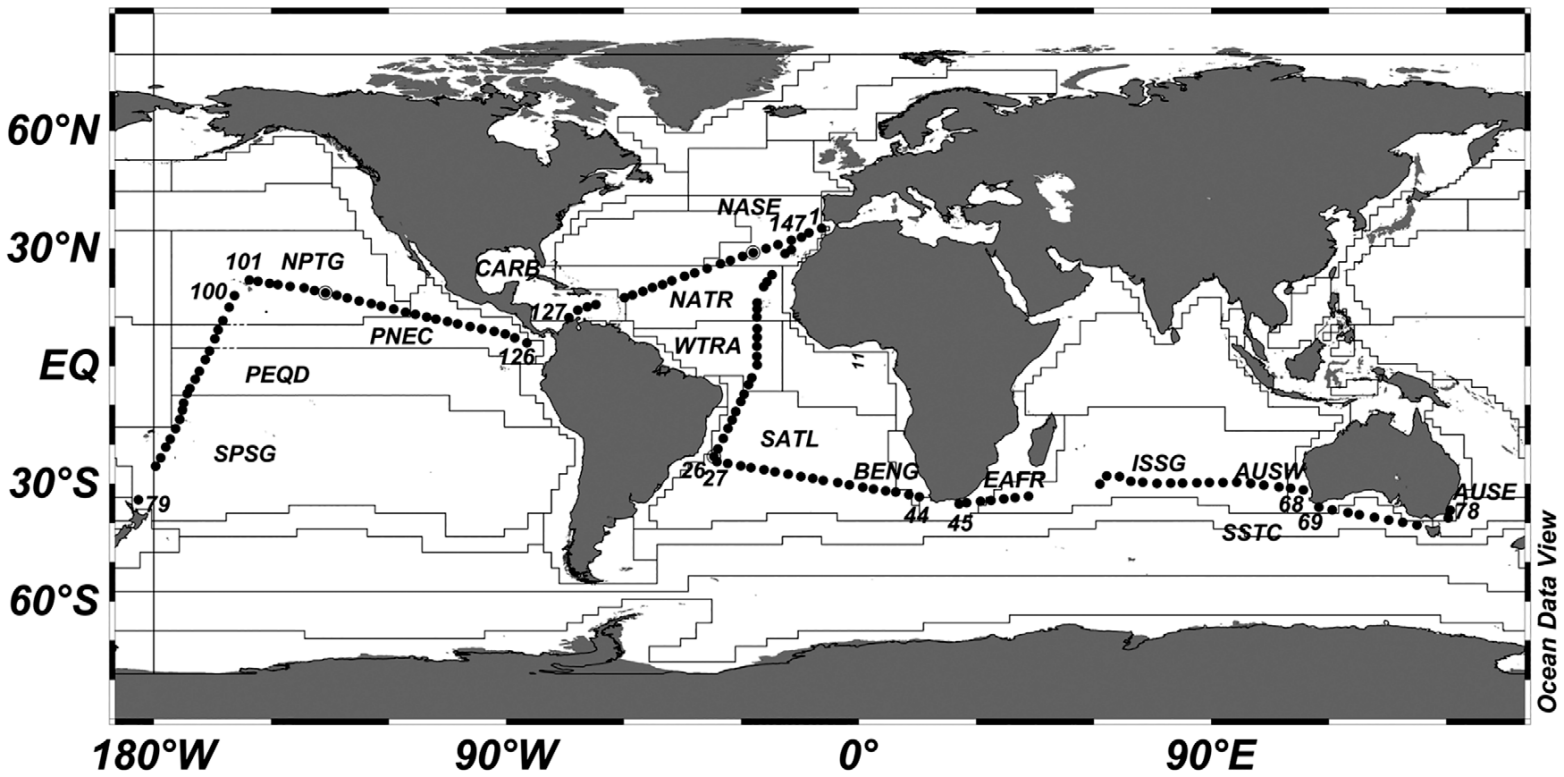
Malaspina-2010 cruise track. The position of the stations and the outline of the Longhurst provinces according to [17] (see Tables 1 and S5) are shown (note that these boundaries are dynamic and that their position in Malaspina-2010 may not coincide exactly with that shown in the figure). The numbers along the tracks indicate the first and last stations of each leg.

The Malaspina-2010 cruise circumnavigated the globe covering tropical, subtropical and temperate oceans between 35°N and 40°S in eight consecutive transects (Fig 1, Tables 1, 2 and S5) between the following stopovers: Cádiz (Spain)—Rio de Janeiro (Brazil)–Cape Town (South Africa)–Perth (Australia)–Sydney (Australia)–Auckland (New Zealand)–Honolulu (Hawaii, USA)–Cartagena de Indias (Colombia)–Cartagena (Spain). To enhance comparability among results from different disciplines, the oceanographic stations visited during the Malaspina-2010 cruise were assigned to different domains and biogeographical provinces based on the classification of Longhurst [16]; the boundaries used in Fig 1 were obtained from [17]. Thus, the cruise track (Tables 1, 2 and S5) crossed successively the following provinces; Leg 1: NE Atlantic Subtropical Gyral (NASE), North Atlantic tropical Gyral (NATR), Western tropical Atlantic (WTRA), South Atlantic Gyral (SATL); leg 2: SATL, Benguela Coastal (BENG); Leg 3: East Africa Coastal (EAFR), Indian Subtropical Gyre (ISSG), Australia-Indonesia Coastal (AUSW); Leg 4: Australia-Indonesia Coastal (AUSW), South Subtropical Convergence (SSTC), East Australia Coastal (AUSE); Leg 5: South Pacific Subtropical Gyre (SPSG), Equatorial Pacific Divergence (PEQD), North Pacific Equatorial Countercurrent (PNEC), North Pacific Tropical Gyre (NPTG); Leg 6: NPTG, PNEC; leg 7: Caribbean Sea (CARD), NATR and NASE. The last stations of leg 6 (122–126), within the PNEC, took place in the Costa Rica (or Mesoamerican) Dome, a region of enhanced biological productivity [18]. Most samples were taken in international waters. For research operations in exclusive economic zones, permission was requested from the governments of the corresponding countries. Sampling did not involve endangered or protected species.

**Table 1:** Malaspina-2010 cruise schedule.

Leg	Beginning (Place, date)	End (Place, date)	Province/ Stations
1	Cádiz, 14-12-2010	Rio de Janeiro, 13-01-2011	NASE/ 1–4
			NATR/ 5–10
			WTRA/ 11–18
			SATL/ 19–26
2	Río de Janeiro, 17-01-2011	Cape Town, 6-02-2011	SATL/ 27–41
			BENG/ 42–44
3	Cape Town, 11-02-2011	Perth, 13-03-2011	EAFR/ 45–47
			ISSG/ 48–65
			AUSW/ 66–68
4	Perth, 17-03-2011	Sydney, 30-03-2011	AUSW/ 69
			SSTC/ 70–76
			AUSE/ 77–78
5	Auckland, 16-04-2011	Honolulu, 8-05-2011	SPSG/ 79–89
			PEQD/ 90–97
			NPTG/ 98–100
6	Honolulu, 10-0-2011	Cartagena de Indias,10-06-2011	NPTG/ 101–115
			PNEC/ 116–126
7	Cartagena de Indias, 19-06-2011	Cartagena, 14-07-2011	CARB/ 127–130
			NATR/ 131–139
			NASE/ 140–147

Dates, start and end locations, provinces and stations covered during each leg. The province assignation follows [16], with some modifications as reported in http://metamalaspina.imedea.uib-csic.es/geonetwork/srv/en/main.home. See Table 2 for abbreviations.

**Table 2:** Provinces and domains visited during the Malaspina-2010 cruise*.

Provinces	Province abbreviation	Province Code	Domains	Domain code
East Australian Coastal	AUSE	E	Coastal	C
Australia-Indonesia Coastal	AUSW	U	Coastal	C
Benguela Current Coastal	BENG	B	Coastal	C
Caribbean	CARB	R	Trades	T
East Africa Coastal	EAFR	A	Coastal	C
Indian South Subtropical Gyre	ISSG	I	Trades	T
North Atlantic Subtropical Gyre Province East	NASE	N	Westerlies	W
North Atlantic Tropical Gyre	NATR	T	Trades	T
North Pacific Tropical Gyre	NPTG	G	Trades	T
Pacific Equatorial Divergence	PEQD	Q	Trades	T
North Pacific Equatorial Countercurrent	PNEC	C	Trades	T
South Atlantic Gyral province	SATL	S	Trades	T
South Pacific Subtropical Gyre Province	SPSG	P	Westerlies	W
South Subtropical Convergence Province	SSTC	Y	Westerlies	W
Western Tropical Atlantic Province	WTRA	W	Trades	T

*Names and abbreviations according to [16].

### Hydrography and sampling

In general, two vertical profiles of Conductivity-Temperature-Depth (CTD) were carried out at a fixed position every day, a first one down to 4000 m depth at 5:00 and a second one, starting around 10:00 local time, down to 200 m depth. The CTD, a SeaBird 9/11-plus, was equipped with dual conductivity and temperature sensors, calibrated at the SeaBird laboratory before the cruise. Water samples were obtained using a rosette of 24 10-liter Niskin bottles. Profiles of underwater photosynthetically active radiation (PAR) were obtained with a 4π Biospherical QCP2300-HP sensor attached to the CTD. The mixed layer depth (MLD) was defined [19] as the first depth (z) where σ_θ_(z)- σ_θ_(10)> 0.125 kg m^-3^, where σ_θ_(z) and σ_θ_(10) are, respectively, the potential density anomalies at depths z and 10 m. The Ocean Data View software [20] was used to present the distribution of hydrographical variables.

Water samples for nutrient and total Chl *a* determination were collected from about 10 depths between surface and 200 m, including those selected for phytoplankton sampling. Water for fractionated Chl *a* analyses and for phytoplankton examination was taken from the Niskin bottles of the second cast of the rosette, at the depth of the 20% light level and at the depth of the subsurface chlorophyll *a* (Chl *a*) maximum (SCM). Additional surface seawater samples (3 m depth) were collected with a 30 L Niskin bottle. In total, 406 phytoplankton samples were processed.

### Phytoplankton analysis

Approximately 250 cm^3^ of water were placed in a glass bottle and fixed with hexamine-buffered formaldehyde solution (4% final formalin concentration). A 100 cm^3^ composite chamber was filled with sample water and its content was allowed to settle for 48 hours. At least two transects of the chamber bottom were observed with an inverted microscope [21] at 312 X magnification to enumerate the most frequent, generally smaller, phytoplankton forms. Additionally, the whole chamber bottom was examined at 125 X magnification to count the larger, less frequent cells. In both cases, all cells encountered were tallied. Classification was done at the genus or species level when possible, but many taxa could not be identified and were pooled in categories such as “small flagellates” or “small dinoflagellates”; references to the literature used can be found in [22–38].

Note that the inverted microscope technique is not adequate for cells in the picoplankton size range, which may not sediment and deteriorate easily in fixed samples, and that checklists must be interpreted with caution, because of the limitations inherent to morphotypic phytoplankton identification.

### Chl *a* and inorganic nutrient determinations

To determine total Chl *a* concentration [39], a volume of water ranging between 200 and 500 cm^3^ was filtered through GF/F glass fibre filters that were subsequently frozen at -20°C and, after a minimum of 6 hours, introduced in acetone 90% and left for 24 hours in a refrigerator, in the dark. The fluorescence of the acetonic extracts was determined with a Turner Designs fluorimeter calibrated with a Chl *a* standard (Sigma-Aldrich); no phaeopigment correction was applied. Chl *a* concentration for different size fractions was obtained by sequential filtering of an additional 500 cm^3^ water sample through Poretics (polycarbonate) membrane filters of pore sizes 20 μm, 2 μm and 0.2 μm. Total Chl a values are those of the GF/F filters; however, as these filters tended systematically to collect more Chl *a* than 0.2 μm membrane filters, the proportion of Chl *a* in a particular size fraction was referred to the total obtained by adding up the Chl *a* collected in the three consecutive membrane filters. Dissolved inorganic nutrients were analysed with a Skalar AutoAnalyzer, using the procedures of Grasshoff et al. [40], as described in [41]. The nitracline (starting) depth was defined by visual inspection as the shallowest depth at which concentrations of nitrate began to increase consistently; when nitrate concentration at surface was ≥ 1.5 μmol L^-1^, the nitracline depth was considered to be 0 m. In general, the nutriclines of silicate and phosphate coincided with that of nitrate, although sometimes they started at different depths. The nitracline bottom was assumed to be 200 m (or the closest depth with measurements if this depth was not available).

### Statistical analyses

The composition of the phytoplankton was summarized by means of a principal component analysis (PCA) [42] based on the correlation matrix among log-transformed abundances of the 76 taxa that were present in more than 60 samples (about a 15% of the total), including phytoplankton and ciliates (Table 3, S1 Appendix). The logarithmic transformation of an abundance *x* was performed as *x*’ = log(*x*+10); the number 10 was used instead of 1 because 10 (cells L^-1^) was the smallest number recorded in the data set. Various PCA were carried out with different taxa selection criteria (for example, including only 79 well-defined taxa that were present at least 20 times); a tridimensional non-metric multidimensional scaling (NMDS) analysis using the Bray-Curtis distance was also applied to the 76 taxa selected for the PCA. As all these analyses gave globally similar results, the comments in the following sections will be centred in the 76-taxa PCA. The software packages used included Systat 11 and PRIMER 5 (Plymouth Routines in Multivariate Ecological Research).

**Table 3:** Names and statistical information (referred to samples with non-zero abundance) of the taxa included in the principal component analysis.

	Times present	Max.	Mean ± SD
DINOFLAGELLATES			
*Gyrodinium* spp.	374	380	70 ± 61
*Torodinium robustum*	362	340	55 ± 47
*Lessardia elongata*	350	548	41 ± 44
*Oxytoxum variabile*	309	1233	44 ± 83
*Oxytoxum minutum*	263	190	32 ± 26
*Cochlodinium* spp.	251	100	24 ± 18
*Scrippsiella* spp.	246	100	22 ± 17
Dinoflagellate cysts	232	90	19 ± 12
*Oxytoxum* spp.	194	70	17 ± 11
Unid. dinoflagellates (with inclusion bodies)	146	200	42 ± 39
*Protoperidinium* spp.	131	80	15 ± 10
*Pronoctiluca acuta*	110	190	16 ± 18
Tripos teres*	107	50	16 ± 9
*Oxytoxum coronatum*	104	180	18 ± 21
*Gymnodinium* spp (20–40 μm)	99	70	17 ± 11
*Gymnodinium* spp. (> 40 μm)	95	60	16 ± 10
*Micracanthodinium claytonii*	88	50	15 ± 8
*Oxytoxum scolopax*	86	50	13 ± 6
*Podolampas spinifer*	84	30	12 ± 5
*Oxytoxum mediterraneum*	79	40	14 ± 7
*Gymnodinium* sp. ("pumpkin")	78	60	17 ± 12
*Paleophalacroma unicinctum*	77	30	12 ± 5
*Oxytoxum longiceps*	71	40	13 ± 6
Tripos fusus*	65	60	13 ± 8
*Dinophysis* spp. (small, rounded)	64	40	13 ± 6
*Gonyaulax* spp.	62	20	11 ± 2
Unid. dinoflagellates (large)	406	1520	311 ± 213
Unid. dinoflagellates (small, < 20 μm)	405	9042	2110 ± 1397
DIATOMS			
Unid. pennate diatoms ("benthic-like", large)	366	7124	134 ± 420
Unid. pennate diatoms (small, < 20 μm)	344	2466	202 ± 321
*Leptocylindrus mediterraneus* (with *Rhizomonas setigera*)	201	1620	76 ± 130
Unid. pennate diatoms	201	960	67 ± 142
*Thalassiosira* spp.	200	4250	111 ± 426
*Pseudo-nitzschia* spp	125	4420	303 ± 627
*Rhizosolenia* spp.	123	950	49 ± 110
Pennate diatom (sp. 2, "spindle-like")	116	130	18 ± 17
Unid. centric diatoms	100	240	23 ± 28
*Chaetoceros* spp. (<20 μm)	92	1918	135 ± 303
*Mastogloia rostrata*	87	240	23 ± 32
*Hemiaulus hauckii*	84	6713	318 ± 1104
*Planktoniella sol*	80	140	34 ± 31
COCCOLITHOPHORES			
Unid. coccolithophores (small, < 10 μm)	406	48675	6577 ± 6795
Unid. coccolithophores (large)	401	1781	216 ± 207
*Discosphaera tubifera*	301	3014	299 ± 353
*Syracosphaera pulchra* HET	295	250	46 ± 42
*Umbellosphaera irregularis*	261	8631	686 ± 924
*Syracosphaera pulchra* HOL	212	310	48 ± 57
*Rhabdosphaera clavigera*	206	190	29 ± 26
*Helicosphaera carteri*	199	190	26 ± 28
*Calciosolenia brasiliensis*	196	2329	110 ± 271
*Ophiaster hydroideus*	185	3699	326 ± 589
*Calcidiscus leptoporus*	150	1644	124 ± 235
*Umbilicosphaera sibogae*	146	740	111 ± 168
*Calciosolenia murrayi*	133	7480	175 ± 705
Coccolithophore (sp. 1, "*Coronosphaera*-like")	133	280	23 ± 28
*Acanthoica quattrospina*	94	959	29 ± 105
Coccolithophore (sp. 4, "dark", 11–15 μm)	91	150	31 ± 30
*Algirosphaera robusta*	85	1781	143 ± 264
*Umbilicosphaera anulus*	80	4521	225 ± 626
*Michaelsarsia elegans*	74	685	34 ± 92
*Syracosphaera prolongata*	67	411	25 ± 52
*Calciopappus rigidus*	66	680	33 ± 83
*Oolithotus* spp.	60	1644	107 ± 235
Syracosphaera spp.	61	80	21 ± 15
OTHER PHYTOPLANKTON			
Cryptomonads	121	1096	196 ± 206
*Dictyocha fibula*	160	480	34 ± 56
*Halosphaera viridis* (phycoma)	76	150	25 ± 25
Colonial flagellate (sp. 1, colonies)	120	190	27 ± 28
*Pterosperma moebii*	116	630	70 ± 110
*Trichodesmium* sp. (filaments)	71	1730	188 ± 339
Unid. nanoflagellates (3–20 μm)	406	168784	8025 ± 12123
CILIATES			
Ciliates ("naked", <30 μm)	396	4760	159 ± 328
Ciliates ("naked", > 30 μm)	390	940	110 ± 107
*Strombidium* spp.	345	510	67 ± 70
Tintinnids (large)	355	200	41 ± 35
Tintinnids (< 40 μm)	117	200	17 ± 20

Unid. = Unidentified. Abundances in cells L^-1^, Max. = maximum, SD = standard deviation. Minimum abundances were almost always 10 cells L^-1^ (one cell in the whole chamber after settling 100 mL, or 10 cells in 1 L); the exceptions were unidentified dinoflagellates (large and small), coccolithophores and nanoflagellates, which presented minimum abundances ranging between 20 and 410 cells L^-1^. Nanoplankton was identified at 312 X. The less abundant microplankton forms were counted at 125 X, but identification was checked at 312 x when necessary.

* Formerly, genus *Ceratium* [30].

## Results

### Global phytoplankton distribution

**Fig 2:**
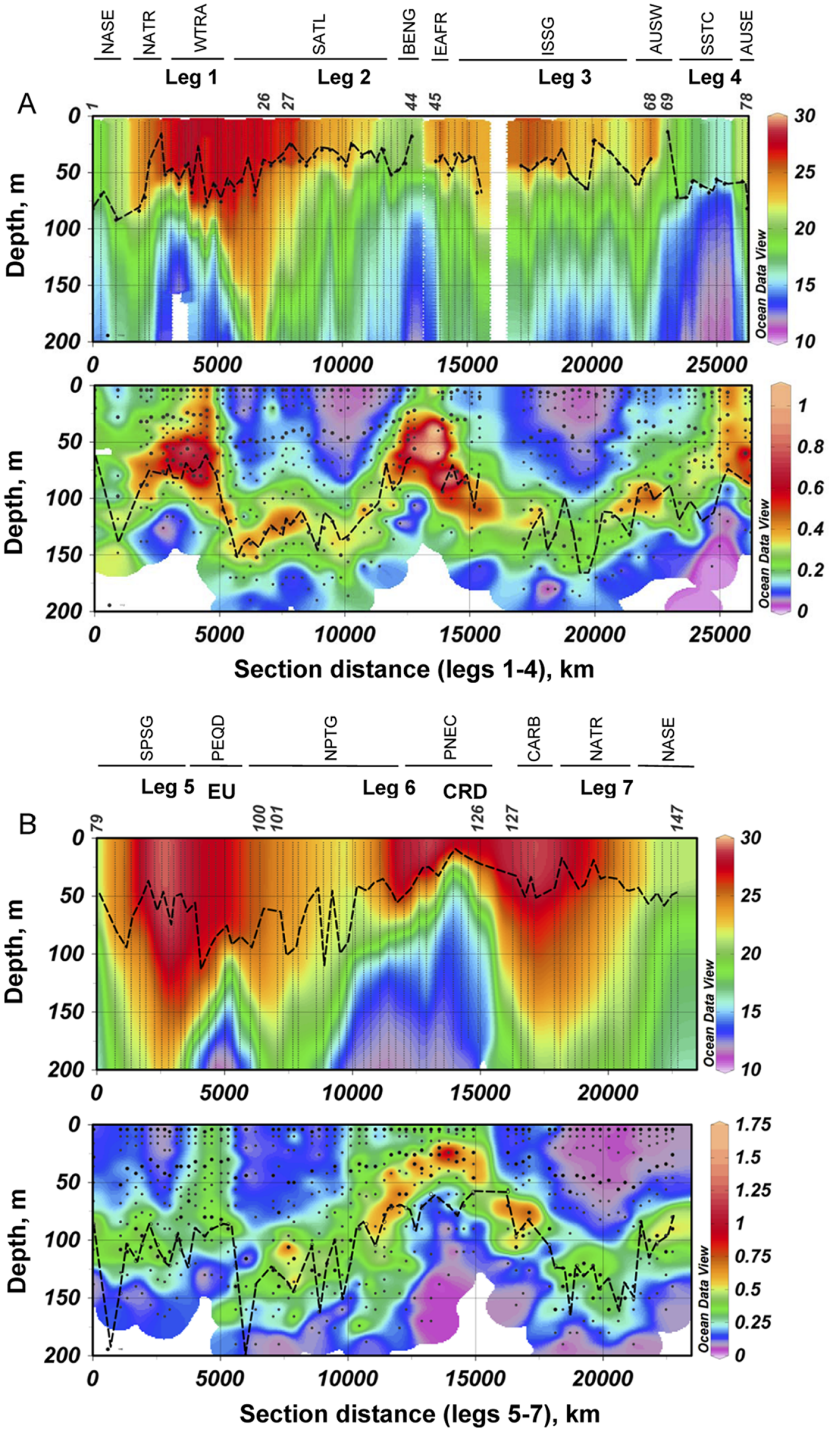
Distributions of temperature and Chl *a* concentration along the Malaspina-2010 cruise. (A) Temperature (°C). (B) Chl *a* (mg m^-3^). The dashed lines indicate the mixed layer depth in (A) and the 1% light level in (B). The dots in (B) indicate the Chl *a* sampling depths; the larger dots correspond also to phytoplankton samples. The different legs and the provinces crossed are shown on top of the figure. EU = Pacific Equatorial Upwelling, CRD = Costa Rica (or Mesoamerican) Dome. The numbers of the initial and final station of each leg are indicated on top of the temperature graph.

In correspondence with the cruise track, which crossed mainly oligotrophic tropical and subtropical regions, and the late spring-summer timing of the expedition, most stations presented a stratified water column with an upper mixed layer and a marked pycnocline (Fig 2A). The mixed layer was nutrient-depleted (data not shown) and its depth (MLD) was in general shallower than the 1% light level. With the exception of several stations in the Pacific Equatorial Divergence region (PEQD), a subsurface Chl *a* maximum (SCM) was found at approximately the 1% light level, at depths from about 30 m in the Costa Rica Dome down to 160 m in the tropical regions of the Atlantic and the Pacific oceans (Fig 2B). Chl *a* concentration ranged from 0.03 to 0.69 μg L^-1^ at 3 m, from 0.05 to 1.08 μg L^-1^ at the 20% light level and from 0.11 to 1.92 μg L^-1^ at the SCM Note that sometimes the SCM bottle would not close precisely at the actual SCM depth; the minimum Chl *a* value of 0.11 μg L^-1^ corresponds to station 69, at 80 m depth; in this case, the SCM bottle hit a thin (10–15 m) layer of relatively low salinity and low Chl *a* that crossed through the SCM; the Chl a concentration for the SCM peak (estimated from the *in vivo* fluorescence record) would have been closer to 0.19 μg L^-1^. On average, the > 2 μm size fractions accounted for (mean ± standard deviation) 43% ± 14%, 45% ± 14% and 34% ± 15% of total Chl *a* at 3 m, 20% light level and SCM depths, respectively. The corresponding proportions for the > 20 μm size fraction were, respectively 10% ± 8%, 10% ± 10% and 7% ± 5%. The highest Chl *a* concentrations were found in some coastal areas near South Africa and Australia, in zones influenced by upwelling or divergences in the Equatorial provinces of the Pacific (PEQD) and Atlantic (WTRA), and in the Costa Rica Dome (PNEC). Generally, these regions presented enhanced fluxes of nitrate (and presumably also of other nutrients) towards the euphotic layer [43] and for simplicity will be collectively designed here as Upwelling- Divergence (U-D) regions. Even in the high Chl a areas, the highest Chl *a* concentrations tended to be at subsurface levels, with the exception of stations like those of the Pacific Equatorial Upwelling, in which there was no clear SCM. These stations tended also to have a higher proportion than the global average of > 2 μm Chl *a* (53% ± 11%, 54% ± 6% and 46% ± 13% for 3 m, 20% light level and SCM depths). The poorest stations were found in the Atlantic and Indian Oceans and tended to have a pronounced SCM.

Overall, 403 taxa (including several ciliates and other heterotrophic protists, various cysts and fungal spores) were recorded in 406 samples (the full data set is stored in the Digital Malaspina-2010 database, http://metamalaspina.imedea.uib-csic.es/geonetwork/srv/en/main.home, search term “Phytoplankton sampling from Niskin bottles”). A summary of descriptive statistics for the 76 taxa that occurred at least in 60 samples and were included in the PCA is shown in Table 3, and distribution maps of some taxa positively or negatively correlated with the components is presented in S1–S4 Figs In the 403 taxa data set, dinoflagellates, including autotrophic, mixotrophic and heterotrophic forms, presented the largest number of taxa (242), followed by diatoms (72) and coccolithophores (13). The highest population densities corresponded to pooled categories like “Unidentified nanoflagellates (3–20 μm)”, “Unidentified coccolithophores (small, < 10 μm)” (mostly *Emiliania huxleyi* and *Gephyrocapsa* spp.), and “Unidentified dinoflagellates (small, < 20 μm)”. Among the identified coccolithophore species, the most abundant were *Umbellosphaera irregularis*, *Discosphaera tubifera*, *Ophiaster hydroideus*, *Calciosolenia murrayi*, *Calciosolenia brasiliensis* and *Calcidiscus leptoporus*. The most abundant among the dinoflagellate taxa that could be attributed to genus or species were *Gyrodinium* spp., *Torodinium robustum*, *Lessardia elongata*, *Oxytoxum variabile* and *Oxytoxum minutum*. A variety of large forms belonging to genera like *Tripos* (formerly *Ceratium*), *Ornithocercus* and *Histioneis* were found infrequently in settled inverted microscope samples and were not included in the 76 taxa subset used for the PCA analysis, but were well represented in phytoplankton net hauls (data not shown). The globally most abundant diatom genera and species were *Pseudo-nitzschia* spp, *Hemiaulus hauckii* (with its cyanobacterial symbiont *Richelia intracellularis*), *Leptocylindrus mediterraneus* (with the flagellate *Solenicola setigera*), small *Chaetoceros* spp. (< 20 μm), *Rhizosolenia* spp. (many of them with *Richelia intracellularis*) and *Planktoniella sol*. Ciliates were mainly represented by unidentified aloricate forms and *Strombidium* spp. Other taxa found in the samples were non-calcifying haptophytes like *Phaeocystis* spp. and *Chrysochromulina* spp., silicoflagellates, cryptomonads, phycomes of the prasinophytes *Pterosperma* spp. and *Halosphaera viridis*, a “Colonial flagellate (sp. 1, colonies)” and the cyanobacterial genus *Trichodesmium*. *Phaeocystis* spp. and *Chrysochromulina* spp.were not included in the 76 taxa data set because the number of samples in which they could be reliably identified did not reach the frequency threshold. The “Colonial flagellate (sp. 1, colonies)” presented globular colonies of 10–20 chlorophyll-containing cells (each about 12–14 μm in diameter) with single long flagella and was counted as colonies. Some of the species excluded from the multivariate analysis were abundant in particular areas; for example, *Brachidinium capitatum*, with 35 occurrences and an average (when present) of 114 cells L^-1^ reached 3570 cells L^-1^ at station 45, 40 m depth (EAFR province), and *Asterionellopsis glacialis*, found only once, at the same station but at 60 m depth, presented 820 cells L^-1^. However, neither these species [44,45] nor the other discarded taxa could be considered as province-characterising.

**Fig 3:**
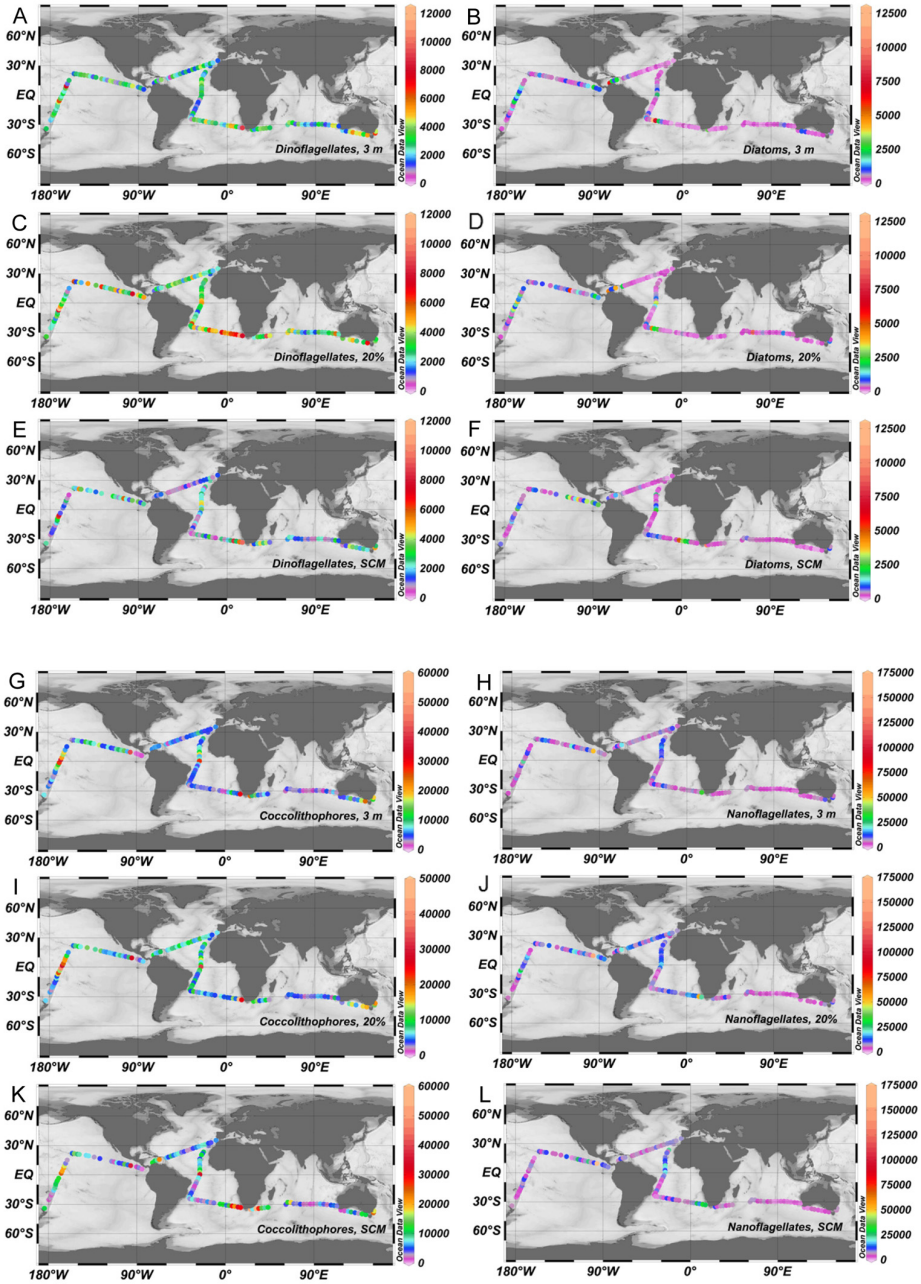
Distribution of major phytoplankton groups (cells L^-1^). (A, C, E) Dinoflagellates. (B, D, F) Diatoms. (G, I, K) Coccolithophores. (H, J, L) “Unidentified nanoflagellates (3–20 μm)”. (A, B, G, H) 3 m depth. (C, D, I, J) 20% light level. (E, F, K, L) SCM.

**Fig 4:**
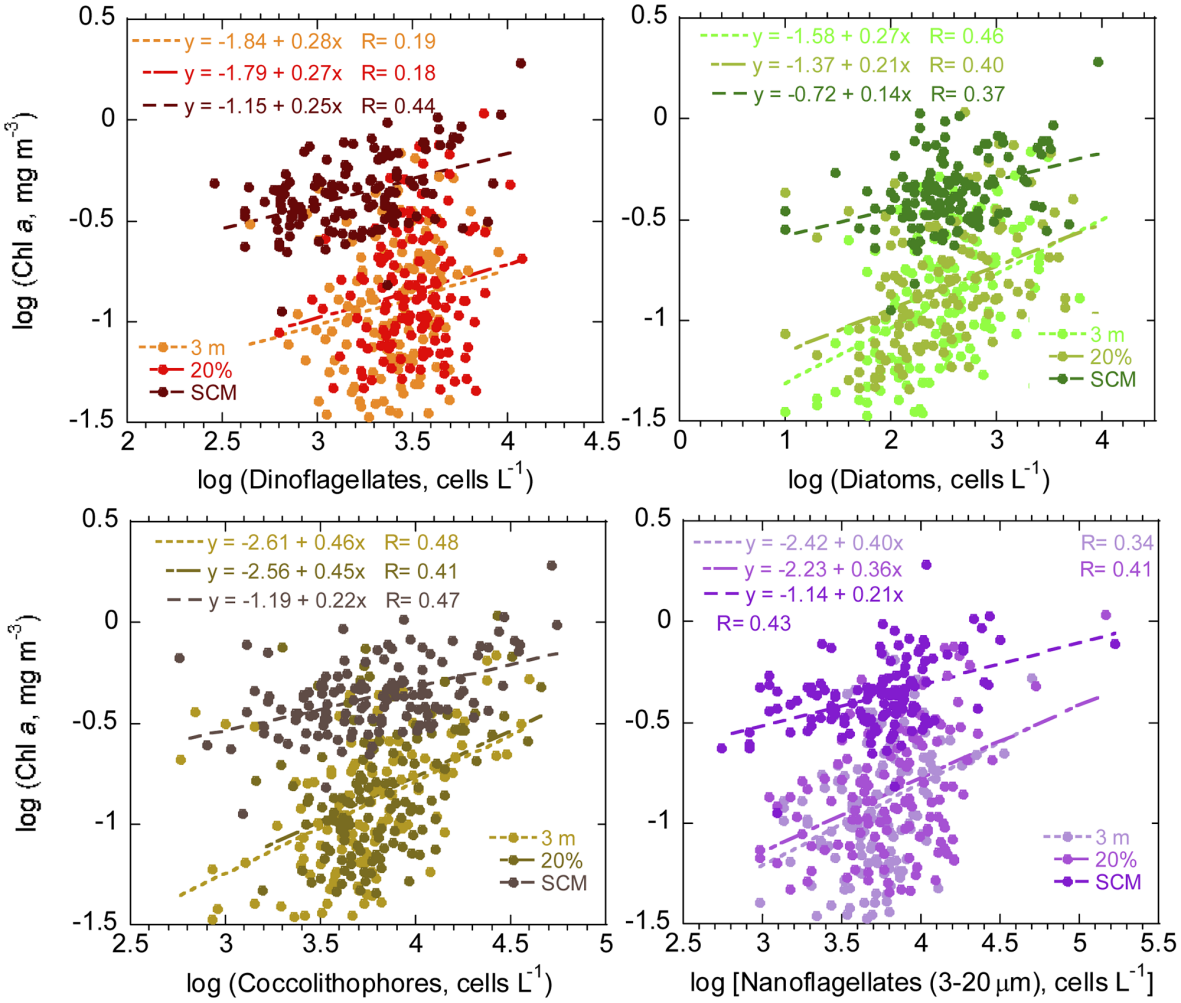
Relationships between abundance of major phytoplankton groups (cells L^-1^) and Chl *a* (mg m^-3^) concentration. Top left, dinoflagellates. Top right, diatoms. Bottom left, coccolithophores. Bottom right, “Unidentified nanoflagellates (3–20 μm)”. The three sampling depths (3 m, 20% light level and SCM) are indicated by different colours. The corresponding regression lines (dashed) and equations are indicated.

“Unidentified nanoflagellates (3–20 μm)” coccolithophores and diatoms presented a background of relatively low population density with a few high points coinciding with the U-D regions (Fig 3); differences in global vertical averages were not significant for these groups (Table 4). Dinoflagellates showed a fairly patchy distribution (Fig 3), with the highest abundances (Table 4) at the 20% light level, followed by those at surface and the SCM depth (Kruskal-Wallis, p< 0.001; Dwass-Steel-Critchlow-Fligner test, p≤ 0.001 for all comparisons). The log-log relationship between major phytoplankton group abundance and total Chl *a* (as determined through GF/F filtration) at different depths exceeded the 0.05 significance level in all cases; regression slopes and intercepts were similar for samples from 3 m and the 20% light level, but slopes were lower and intercepts higher for the SCM (Fig 4). Explained variances (R^2^) ranged from 3% (dinoflagellates at the 20% light level) to 23% (coccolithophores at 3 m depth). Multiple linear regression of log (Chl *a*) on the log-transformed abundance of diatoms and coccolithophores as independent variables raised the explained variance to 36%, 25% and 27% for 3 m, 20% and SCM samples, respectively (n = 133–129). These values increased only marginally (to 37%, 28% and 30%, respectively) when the independent variables included also the log-transformed abundances of dinoflagellates and nanoflagellates.

**Table 4:** Average values of selected variables for the three nominal sampling levels.

	Surface	20% light level	SCM
Depth	3	37 ± 10	101 ± 32
Chl *a*	0.16 ± 0.13	0.19 ± 0.17	0.47 ± 0.23
Percentage of > 2 μm Chl *a*	43 ± 14	45 ± 14	34 ± 15
Dinoflagellates	2828 ± 1368	3538 ± 1805	2055 ± 1771
Diatoms	715 ± 1407	704 ± 1191	665 ± 1096
Coccolithophores	7280 ± 6606	8124 ± 6881	8750 ± 9084
Unid. nanoflagellates (3–20 μm)	6721 ± 5717	9403 ± 13670	8072 ± 15174

Sampling depth in m, Chl *a* concentration in mg m^-3^ and abundance of major phytoplankton groups in cells L^-1^. Average values ± standard deviation (except or the surface depth, which was always 3 m). SCM = Subsurface chlorophyll maximum. Unid. = unidentified. Number of observations (with both Chl *a* and phytoplankton data): surface, 134–138; 20% light level, 128–132; SCM, 132–134.

### Principal component analysis

**Fig 5:**
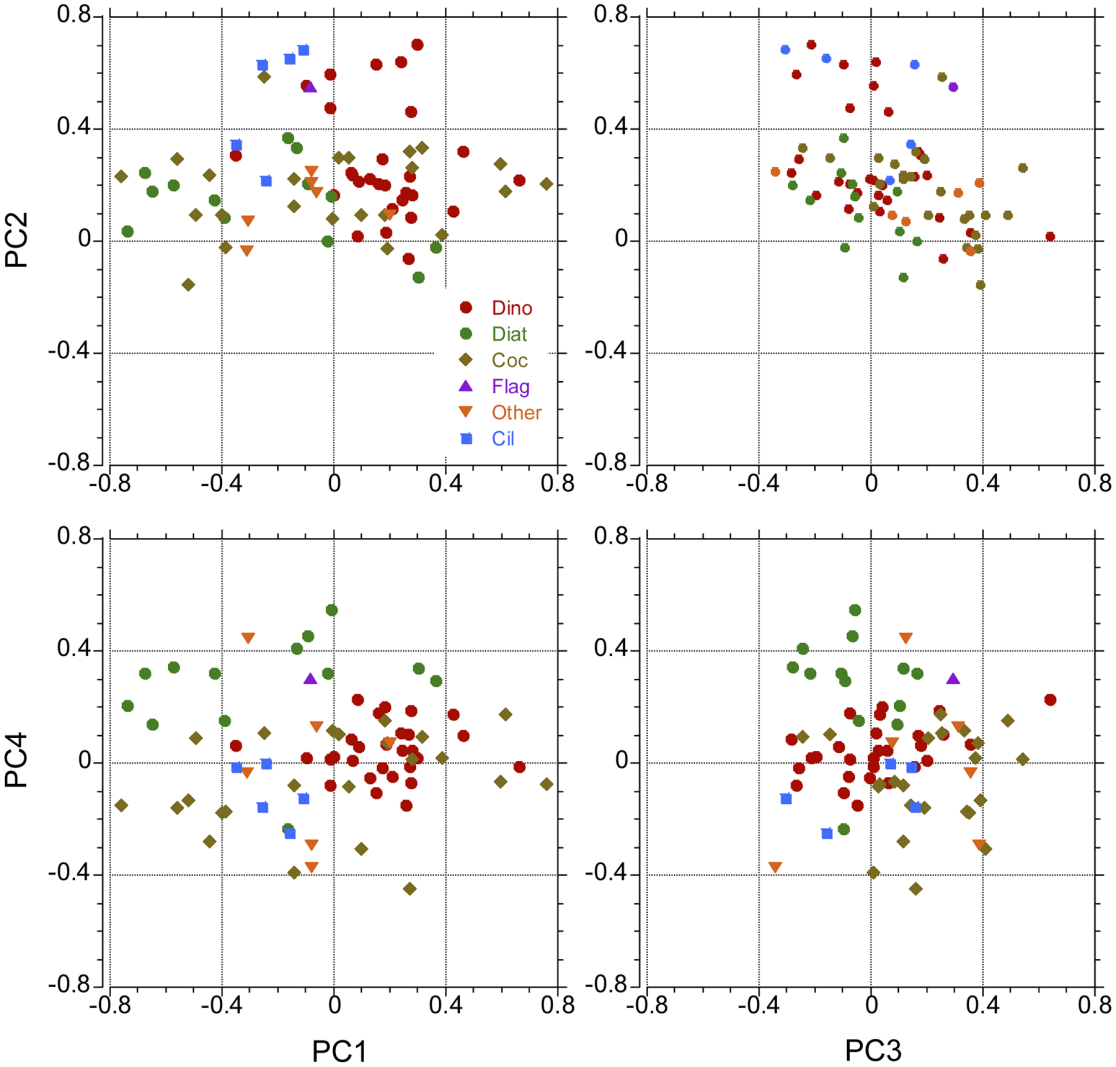
Position of the extremes of the taxa vectors in the space of the principal components. (A) PC1 and PC2. (B) PC3 and PC2. (C) PC1 and PC4. (D) PC3 and PC4. Legend: Dino = Dinoflagellates, Diat = Diatoms, Coc = Coccolithophores, Flag = “Unidentified nanoflagellates (3–20 μm)”, Other = Other taxa, Cil = Ciliates.

The first four principal components (PC1 to PC4) of the PCA, which explained, respectively, 11.3%, 9.1%, 5.3% and 4.2% of the 76-descriptor data set, were retained for further consideration (S1 Table). The taxa with correlation coefficients (or loadings) ≥ 0.3 in absolute value are listed in Tables 5–8. S1–S4 Figs present the distribution of some representative taxa (not all of them included in Tables 5–8, see explanations of the tables), positively or negatively correlated with the components. PC1 presented (Fig 5A and 5C, S1 Fig, Table 5) strong positive loadings with some dinoflagellate and coccolithophore taxa, and negative loadings with *Ophiaster hydroideus* and other coccolithophores, and with “Unidentified pennate diatoms”, *Thalassiosira* spp., *Pseudo-nitzschia* spp. and *Planktoniella sol*, among other diatoms (note that assignation of positive or negative sign to one or the other extreme of the loading sequence is arbitrary; in general, the side with more descriptors of the same sign is chosen as positive). PC2 (Fig 5A and 5B, S2 Fig, Table 6) was positively correlated with all variables except for a group of eight taxa with weak negative correlations that comprised *Dinophysis* spp. and *Hemiaulus hauckii* (not included in Table 6 because the corresponding correlations did not reach -0.3), while PC3 (Fig 5B and 5D, S3 Fig, Table 7) expressed mainly an opposition between a group of unidentified dinoflagellates and several coccolithophore categories, on the positive side, and a mixed assemblage with a “Colonial flagellate (sp. 1)”, *Gymnodinium* spp. (large < 40 μm and ciliates on the negative side. The diatom *Planktoniella sol* (not included in Table 7) was also negatively correlated with PC3 but with a correlation coefficient (-0.28) weaker than -0.3. PC4 (Fig 5C and 5D, S4 Fig, Table 8) was positively correlated with several diatoms and presented the most negative correlations with some coccolithophores and the “Colonial flagellate (sp. 1)”.

**Table 5:** Names and codes of taxa with loadings on PC1 ≥ 0.3 or ≤ -0.3.

Number	Taxon name	Code	PC1 loading
44	*Discosphaera tubifera*	Co	0.76
5	*Oxytoxum minutum*	Dn	0.66
47	*Syracosphaera pulchra* HOL	Co	0.61
45	*Syracosphaera pulchra* HET	Co	0.60
7	*Scrippsiella* spp.	Dn	0.46
13	*Tripos teres*	Dn	0.43
57	Coccolithophore (sp. 4)	Co	0.39
39	*Mastogloia rostrata*	Dt	0.36
46	*Umbellosphaera irregularis*	Co	0.32
40	*Hemiaulus hauckii*	Dt	0.30
69	*Pterosperma* sp.2	Op	-0.31
67	*Halosphaera viridis* (phycoma)	Op	-0.31
75	Tintinnids (large)	Cl	-0.35
21	*Gymnodinium* sp. ("pumpkin")	Dn	-0.35
59	*Umbilicosphaera anulus*	Co	-0.39
37	Unid. centric diatoms	Dt	-0.39
60	*Michaelsarsia elegans*	Co	-0.40
38	*Chaetoceros* spp. (<20 um)	Dt	-0.43
54	*Calciosolenia murrayi*	Co	-0.44
63	*Oolithotus* spp.	Co	-0.49
58	*Algirosphaera robusta*	Co	-0.52
50	*Calciosolenia brasiliensis*	Co	-0.56
41	*Planktoniella sol*	Dt	-0.57
33	*Thalassiosira* spp.	Dt	-0.65
34	*Pseudo-nitzschia* spp.	Dt	-0.68
32	Unid. pennate diatoms	Dt	-0.74
51	*Ophiaster hydroideus*	Co	-0.76

Unid. = Unidentified

**Table 6:** Names and codes of taxa with loadings on PC2 ≥ 0.3.

Number	Taxon name	Code	PC2 loading
27	Unid. dinoflagellates (large)	Dn	0.70
73	Ciliates (naked, >30 μm)	Cl	0.69
74	*Strombidium spp*.	Cl	0.66
28	Unid. dinoflagellates (<20 μm)	Dn	0.64
72	Ciliates (naked, <30 μm)	Cl	0.63
1	*Gyrodinium* spp.	Dn	0.63
2	*Torodinium robustum*	Dn	0.59
42	Unid. coccolithophores (<10 μm)	Co	0.58
4	*Oxytoxum variabile*	Dn	0.56
71	Unid. nanoflagellates (3–20 μm)	Op	0.55
6	*Cochlodinium* spp.	Dn	0.47
3	*Gymnodinium elongatum*	Dn	0.46
31	*Leptocylindrus mediterraneus*	Dt	0.37
75	Tintinnids (large)	Cl	0.35
29	Unid. penn. diat. (large, "benthic like")	Dt	0.34
46	*Umbellosphaera irregularis*	Co	0.33
7	*Scrippsiella spp*.	Dn	0.32
48	*Rhabdosphaera clavigera*	*Co*	0.32
21	*Gymnodinium* sp. "(pumpkin")	Dn	0.31

Unid. = Unidentified.

**Table 7:** Names and codes of taxa with loadings on PC3 > ≥ 0.3 or ≤ -0.3.

Number	Taxon name	Code	PC3 loading
10	Unid. Dinoflagellates (inclusion bodies)	Dn	0.64
43	Unid. Coccolithophores (large)	Co	0.54
52	*Calcidiscus leptoporus*	Co	0.49
49	*Helicosphaera carteri*	Co	0.41
58	*Algirosphaera robusta*	Co	0.39
66	*Dictyocha fibula*	Op	0.39
64	*Syracosphaera spp*.	Co	0.38
57	Unid. coccolithophore (sp. 4)	Co	0.38
17	*Micracanthodinium claytonii*	Dn	0.36
67	*Halosphaera viridis* (phycoma)	Op	0.36
60	*Michaelsarsia elegans*	Co	0.35
59	*Umbilicosphaera anulus*	Co	0.34
55	Unid. coccolithophore (sp.1)	Co	0.33
65	Cryptomonads	Op	0.31
73	Ciliates (naked, <30 μm)	Cl	-0.30
68	Colonial flagellate (sp. 1)	Cl	-0.34

Unid. = Unidentified.

**Table 8:** Names and codes of taxa with loadings on PC4 ≥ 0.3 or ≤ -0.3.

Number	Taxon name	Code	PC4 loading
36	Pennate diatom (sp. 2, "spindle-like")	Dt	0.55
30	Unid. pennate diatoms (small, "benthic like")	Dt	0.45
69	*Pterosperma moebii*	Op	0.45
29	Unid. pennate diatoms (large, "benthic like")	Dt	0.41
41	*Planktoniella sol*	Dt	0.34
40	*Hemiaulus hauckii*	Dt	0.34
34	*Pseudo-nitzschia* spp.	Dt	0.32
35	*Rhizosolenia* spp.	Dt	0.32
38	*Chaetoceros* spp. (small, <20 μm)	Dt	0.32
71	Unid. nanoflagellates (3–20 μm)	Op	0.30
49	*Helicosphaera carteri*	Co	-0.30
68	Colonial flagellate (sp. 1)	Op	-0.37
62	*Calciopappus rigidus*	Co	-0.39
48	*Rhabdosphaera clavigera*	Co	-0.45

Unid. = Unidentified.

**Fig 6:**
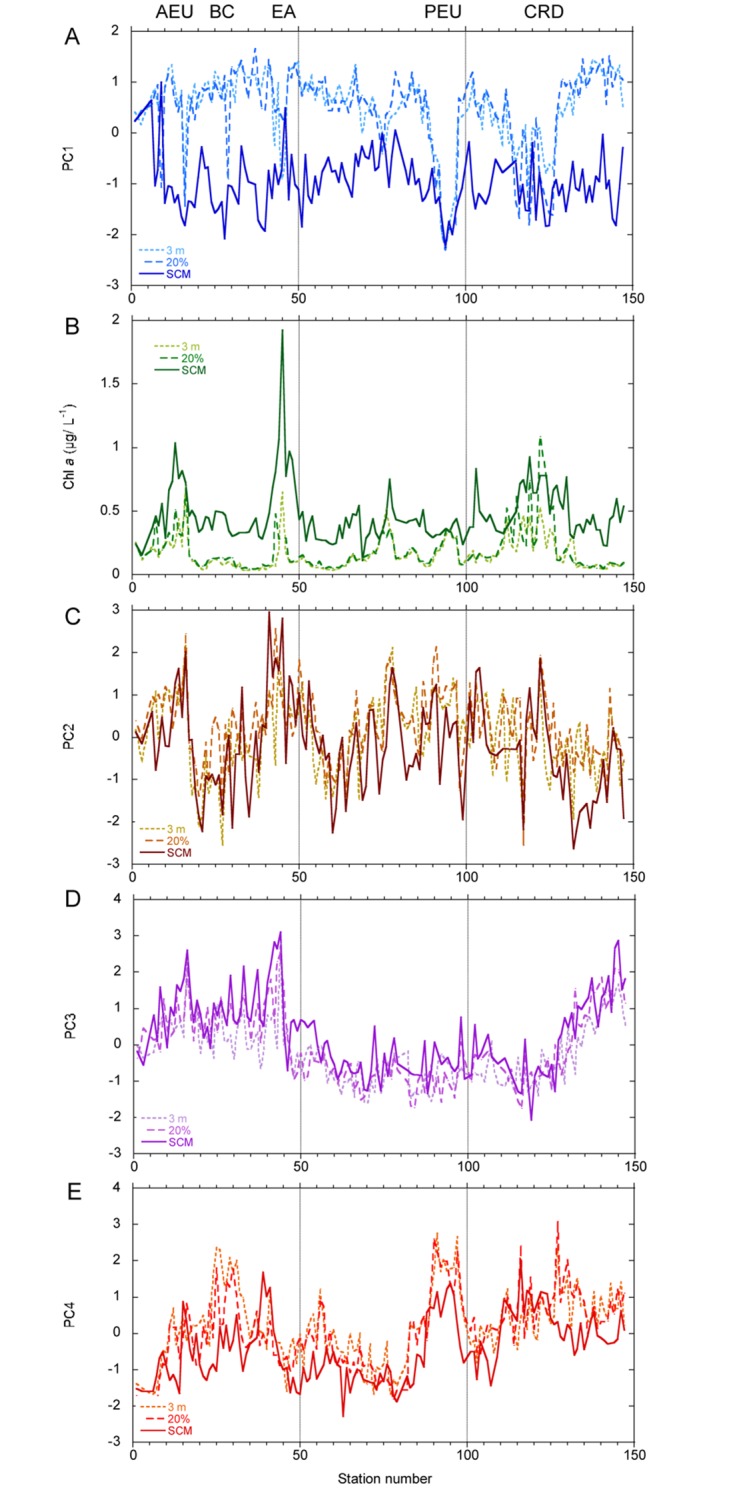
Distribution of the four first principal components and Chl *a* (mg m^-3^) along the Malaspina-2010 cruise. (A) PC1. (B) Chl *a*. (C) PC2. (D) PC3. (E) PC4. The three sampling depths (3 m, 20% light level and SCM) are indicated by different colours and line styles. AEU = Atlantic Equatorial Upwelling, BC = Brazilian Coast, PEU = Pacific Equatorial Upwelling, EA = East Africa, CRD = Costa Rica Dome.

**Fig 7:**
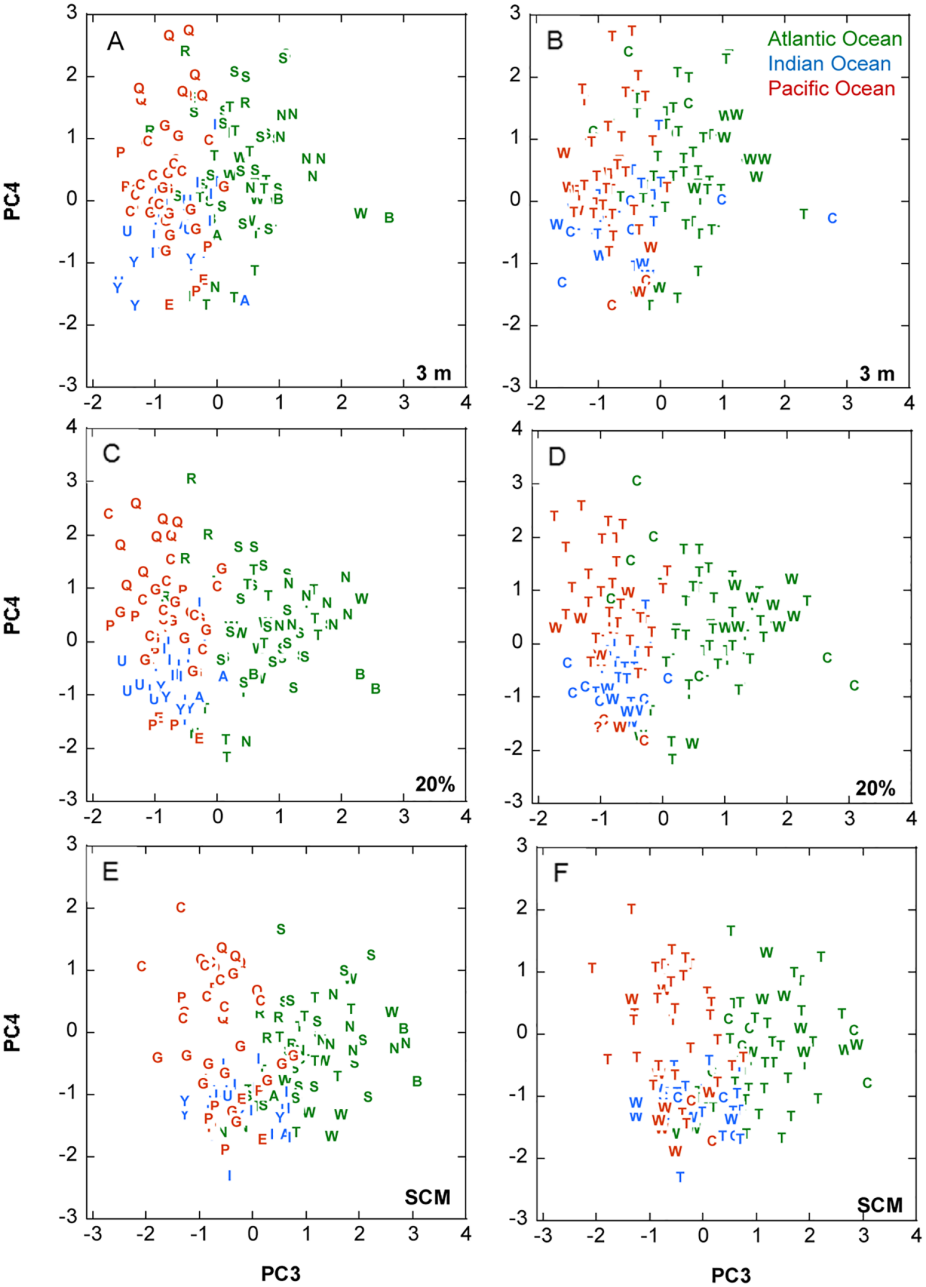
Distribution of the samples in the space of PC3 and PC4. (A, B) 3 m depth. (C, D) 20% light level. (E, F) SCM. In (A, C, E) the letters indicate the provinces and in (B, D, F) the domains (see Table 2 for interpretation).

In general, negative scores of PC1 (Figs 6A and S5) were found at the SCM depth and positive ones at surface and the 20% light level, with the exception of stations of U-D regions (like stations 44–46 near South Africa, 90–97 in the Pacific Equatorial Upwelling and 123–125 in the Costa Rica Dome). As a consequence of this distribution, PC1 presented strong negative and positive global correlations with Chl *a* and PAR, respectively (Table 9). PC2 reflected the distribution of the total cell numbers, dominated by unidentified dinoflagellates, coccolithophores and nanoflagellates, and was therefore positively correlated with the total numbers of all major phytoplankton groups (data not shown) and with Chl *a* at all sampling levels (Fig 6B and 6C, S5 Fig and Table 9). PC3 and PC4 showed only significant correlation with Chl *a* for the 20% light level and for the pooled depths, respectively (Table 9). Positive values of PC3 (Figs 6D, 7 and S6) were generally associated to Atlantic Ocean waters, whereas samples from the Indian and Pacific oceans presented negative scores; in turn, PC4 (Figs 6E, 7 and S6) presented the highest values in surface waters of the Caribbean, in the vicinity of the coast of Brazil and in U-D regions such as the Pacific Equatorial Upwelling, while Indian Ocean samples showed negative PC4 scores. PC4 was also significantly correlated with PAR, both for the whole data set and for the 3 m and SCM depths (Table 9). Some principal components were significantly correlated with temperature or salinity for the whole data set and/or for individual sampling depths (Table 9). However, as discussed below, these correlations should not be taken as indicative of direct causal effects.

**Table 9:** Correlation coefficients of the scores of the four first principal components with selected variables.

Depth range	Principal component	log (Chl *a*, mg m^-3^)	Temperature (°C)	Salinity	PAR^a^ (μmol photon m^-2^ s^-1^)
All depths (n = 360–404)	PC1	-0.75***	0.17**	0.26***	0.32***
	PC2	0.22**	0.02	-0.30***	-0.02
	PC3	0.04	-0.16*	0.61***	-0.07
	PC4	-0.18**	0.51***	-0.08	0.24***
3 m (n = 111–138)	PC1	-0.58***	-0.20*	0.55***	0.09
	PC2	0.46***	-0.17	-0.29**	-0.15
	PC3	-0.18	-0.04	0.63***	0.21*
	PC4	-0.08	0.50***	0.09	0.25*
20% (n = 127–132)	PC1	-0.64***	-0.19	0.51***	0.21*
	PC2	0.52***	-0.09	-0.26**	-0.19
	PC3	-0.29**	-0.13	0.66***	0.22*
	PC4	0.06	0.49***	-0.11	0.07
SCM (n = 132–134)	PC1	-0.22*	-0.35***	0.06	-0.15
	PC2	0.45***	0.09	-0.34***	0.19
	PC3	0.19	-0.40***	0.56***	-0.13
	PC4	0.13	0.36***	-0.19	0.31**

*p<0.01,

**p<0.001,

*** p<0.0001.

^a^ Photosynthetically Available Radiation.

n = number of observations.

Superimposed to the ocean basin gradient represented by PC3, there was often a trend for the samples from the same province to group together, as happened for PEQD (Pacific Equatorial Divergence, “Q”) and CARB (Caribbean, “R”) in the space of PC3 and PC4 (upper left corner of Fig 7A, 7C and 7E, and of S8A, S8D and S8G Fig);. However, there was no clustering of the samples when these were classified by domains (Fig 7B, 7D and 7F).

The three-dimensional NMDS analysis of the same 76-taxa data set gave qualitatively similar results. The relationships between the second NMDS axis and log(Chl *a*) is shown in S7 Fig, and the position of the sample points in the space of the first and second NMDS axes and the relationship between the third NMDS axis and salinity are presented in S9 Fig.

## Discussion

### Global distribution of major phytoplankton groups

Most Malaspina-2010 stations presented stratified water columns (Fig 2), with a wide pycnocline and a nutrient-poor (data not shown) euphotic zone. Chl *a* concentration in the water column presented generally a SCM at depths > 50 m; exception to his pattern were some coastal stations near South Africa and Australia and the U-D regions, in which enhanced nutrient supply allowed the build-up of relatively high Chl *a* concentration in the upper euphotic zone. In agreement with the conceptual model of Margalef [15], under these generally oligotrophic conditions, the phytoplankton was dominated both in abundance and species richness by coccolithophores, dinoflagellates and small flagellates, while diatoms were poorly represented and were only relatively abundant near the coast of Brazil, at the SCM of a few South Atlantic stations (e. g. numbers 38–41) and in upper layers of U-D regions like the Pacific and Atlantic equatorial upwellings and the Costa Rica Dome (Fig 3). The presence of high population densities of dinoflagellates in the upper part of the euphotic zone of nutrient-poor environments has been previously documented [46–48], and can be partly related to the presence of numerous heterotrophic or mixotrophic taxa (features that are difficult to assess with the usual inverted microscope method) and to their ability to perform diurnal vertical migrations that allow them to gather nutrients at deeper levels at night and photosynthesize higher up in the water column during the day. Given a maximum swimming speed for dinoflagellates of near 2 m h^-1^ [49], it is possible that some forms could undertake partial migrations through the water column, for example from the nutricline level so some tens of meters above. However, as sampling time was approximately the same for all stations, we assume that the vertical distributions we found are comparable among them. Mixotrophy is also widespread in other groups, including life stages of coccolithophores and many flagellate forms, and could help to explain the relatively high general abundance of all these organisms throughout the cruise track. In contrast, diatoms presented a pattern of sharp peaks against background concentrations of less than 100 cells L^-1^.

All major phytoplankton groups were positively correlated with Chl *a* (Fig 4), but the variance explained by the multiple linear correlation of log (Chl *a*) on the log-transformed abundances of diatoms and coccolithophores increased only slightly when dinoflagellates and flagellates were added. The low incidence on Chl *a* variability of these last groups could be partly related to the inclusion of heterotrophic forms within them. The decreasing abundance of dinoflagellates with depth and the lack of vertical patterns of the other groups contrast with the downward increase and the marked SCM shown generally by the Chl *a* profiles, a situation reflected in the progressive shift towards higher Chl *a* concentrations of the regression line between Chl *a* and cell abundance, when increasing the sampling depth (Fig 4). As the contribution of nano- and microplanton to total Chl *a* was only about 40%, versus 60% of picophytoplankton, these statistical relationships between Chl *a* and (nano- and micro-) phytoplankton group abundance must also be influenced by variability in picophytoplankton cell numbers and Chl *a* content. In any case, it is likely that the increase of Chl *a* at the SCM was largely due to enhanced Chl *a* content per cell in all phytoplankton size classes, a generic response to photoacclimation to low light and enhanced nutrient availability [50], Cullen [51].

### Trends of variability

Overall, the geographical distribution of major phytoplankton groups agreed with available information. The occurrence of diatoms in the Pacific and Atlantic Equatorial upwellings and near the coast of South Africa has been documented *in situ* and reproduced in satellite and modelling studies [52–55]. Several works [53,55] have also indicated the relatively high abundance of coccolithophores in the Pacific Equatorial upwelling, the Benguela region and near the S and SE Australian coasts.

The first four principal components of the PCA explained 30% of the variance of the 76-species data set, a figure comparable to that found in other phytoplankton studies [47,56]. As can be seen in the distributions of taxa with positive or negative loadings (S1–S4 Figs), the trends of variability detected by these components were due to differences in the relative participation of different taxa rather than to their presence or absence in particular regions. However, our analysis excluded rare taxa (only those present in more than 15% of the samples were selected), a necessary precaution to obtain meaningful correlations [42], and therefore our findings cannot be taken as a support for the idea of “ubiquitous dispersal” of microbial organisms [57]. Furthermore, the apparent worldwide distribution of many species could be in part a result of cryptic and pseudo-cryptic diversity [22], [58], [59]. In addition, several studies have demonstrated that what appeared to be a single taxonomic entity consisted of genetically differentiated strains or species [60,61], a feature that could explain the presence of this entity in different environments. As shown by the comparison of the right and left panels of S7 Fig and the results shown in S9 Fig, the three axes of the NMDS analysis expressed the same general gradients as the first three components of the PCA and will not be considered further.

In order to interpret the potential relationships between biological variables such as Chl *a* concentration, phytoplankton abundance and principal component scores, it is crucial to take into account the sampling structure of the data. The cruise lasted for several months and many variables were affected not only by water mass and geographical variation, but also by the phase of the seasonal cycle at the time of visiting each zone. Seasonal changes may be relatively small [8] in areas like the North Pacific Tropical Gyre (NPTG province), but could be more important at higher latitudes, as can be seen when comparing the first and last stations of the cruise in the NASE Province in the graphs of Fig 6. Malaspina-2010 sampling dates were strongly negatively correlated with salinity (R = - 0.62, -0.61 and -0.62, p << 0.0001, for the 3 m, 20% and SCM sampling depths respectively) as a result of the particular trajectory chosen, which visited most Atlantic stations before those of the less saline Indian and Pacific oceans. This sampling framework and the effect of confounding variables underlie some of the differences in mean variable values and statistically significant correlations found in Tables 9, S2 and S3 Tables, as will be discussed in more detail below.

PC1, which explained the largest fraction of the total variance in our analysis, expressed primarily the contrast between the phytoplankton communities of the upper part of the photic layer (3 m and 20% light level) on the positive score side, and of the SCM on the negative side (Fig 6A); the respectively negative and positive correlations of PC1 with Chl *a* and PAR for the global data set are a consequence of the positive scores of the component in the warmer, well-illuminated surface waters. An apparent exception to this interpretation lies in the large negative scores (Figs 6A and S5A) of Pacific Equatorial Upwelling (PEQD) samples collected not only from the SCM, but also from 3 m and the 20% light level. These samples showed many particular characteristics and were considered separately in S2–S4 Tables. The upper euphotic zone of the PEQD stations contained taxa characteristic of the SCM although with lower abundances (S1C and S1D Fig), a situation that appears to echo the conclusion of Herbland et al. [62] that the seasonal Equatorial Upwelling of the Eastern Atlantic corresponds to the movement towards the surface of the SCM community. PC1 was also strongly negatively correlated with Chl *a* (Table 9) for each individual sampling depth, especially the two upper ones, and with PAR at the 20% light level. Additionally, the stations with the most negative scores of the component presented relatively shallow nitracline depths (S2 and S3 Tables), suggesting that the associated enhancement of nutrient supply into better illuminated levels of the euphotic zone favoured taxa of the deep community. These observations can be compared with those of Estrada [47], who found the same taxa (including several diatom genera) in the upper layers of mixed coastal waters during the winter-spring bloom of the NW Mediterranean and in the SCM during the stratification period. The strong variability associated with the vertical water column gradient agrees with findings of a number of studies carried out in oligotrophic, stratified oceanic water columns [46,48,63,64]. Several taxa detected in this study as strong contributors to the deep or shallow assemblages coincided with those listed in other works. For example, in her analysis of the phytoplankton of the Central North Pacific, Venrick [64] found also that the coccolithophores *Discosphaera tubifera* and *Umbellosphaera irregularis*, and the diatom *Hemiaulus hauckii* were part of the shallow group, while *Calciosolenia murrayi*, species of *Oolithotus* and *Pseudo-nitzschia* spp. belonged to the deep assemblage. Among the diatom taxa of the deep group, *Chaetoceros* spp., *Thalassiosira* spp. and *Pseudo-nitzschia* spp. form patches in the SCM of the Western Mediterranean [47] and *Planktoniella sol* has been cited as a resident of the deeper part of the euphotic zone by Beers et al. [65].

The finding of a principal component, in this case, PC2, positively associated with most of the descriptor variables (the “abundance-richness of taxa” component) is frequent in ecological analyses [47] and reflects the fact that certain sets of ecological conditions tend to be favourable or unfavourable for most species in the community (a situation comparable to that of isometric size in principal component analyses of measures of individuals [66]). PC2 presented a positive correlation with Chl *a* concentration, both for the pooled data and separately for each sampling level (S7 Fig, Table 9); a clear match between the positive peaks of PC2 and Chl *a* occurred (Fig 6B and 6C) in the Atlantic Equatorial Upwelling region (WTRA, stations 12–16) and near the coast of South Africa (BENG and EAFR, stations 42–45). These highly positive PC2 stations tended to present shallower nitraclines and euphotic zone depths (Kruskal-Wallis, p < 0.05) than the strongly negative PC2 stations (S2 and S3 Tables). The 12 variables (Table 6) with the highest positive loadings on PC2 included dinoflagellates, coccolithophores, ciliates and nanoflagellates but no diatoms, indicating that most situations of relatively high phytoplankton abundance in the Malaspina-2010 cruise were associated with taxa from phytoplankton groups characterising advanced phases of succession [15] rather than with the diatom species that tend to dominate seasonal winter and spring blooms. One of the two diatom taxa with loadings exceeding 0.3 was *Leptocylindrus mediterraneus*, which forms a consortium with the heterotrophic flagellate *Solenicola setigera* (which, in turn, as shown by epifluorescence microscopy of fresh Malaspina-2010 samples could be accompanied by potentially diazotrophic picoeukaryotic cyanobacteria), and is ubiquitous in oligotrophic waters [67,68]. The other diatom category with loading > 0.3, the “Unidentified pennate diatoms (large, "benthic like")”, was poorly constrained taxonomically.

**Fig 8:**
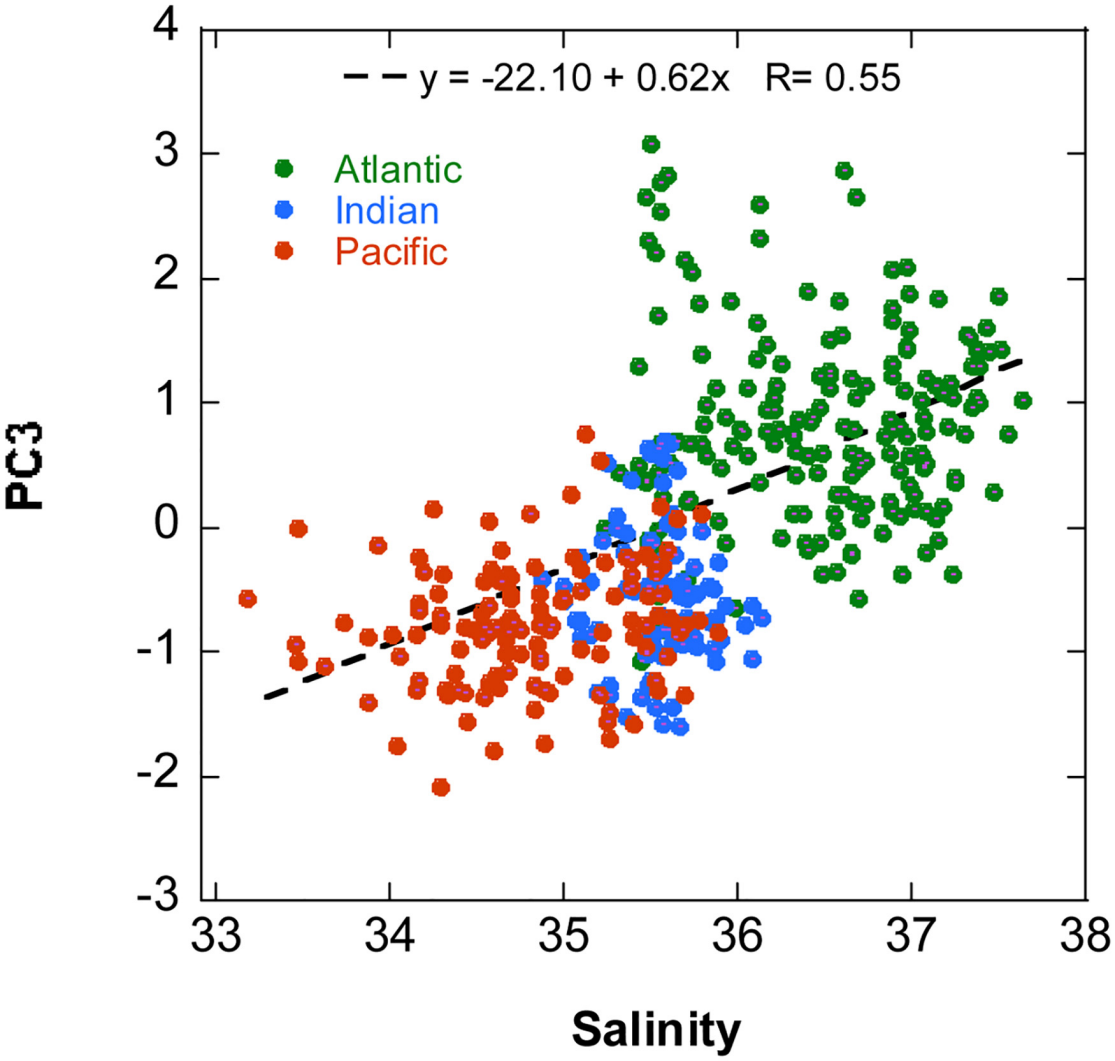
Relationship between salinity and PC3 in different oceans. Green circles, Atlantic Ocean; blue circles, Indian Ocean; red circles, Pacific Ocean. The regression line for the whole data set and the corresponding equation are indicated.

The third principal component, PC3, which separated Pacific and Indian Ocean stations on the negative side from Atlantic stations on the positive one (Figs 6D and 7), represented a biogeographical signature based mostly on the higher importance of several dinoflagellate taxa, large naked ciliates and a colonial flagellate in the Pacific and Indian Oceans, versus that of several coccolithophore species, two dinoflagellate taxa, the silicoflagellate *Dictyocha fibula* and the prasinophyte *Halosphaera viridis* in the Atlantic. High and low PC3 samples (S2 and S3 Tables) presented similar mean MLD and nitracline depth; high PC3 samples tended to come from higher depths than the low PC3 ones, but the component scores were not correlated with PAR for the pooled data set (Table 9). The significant positive correlation of PC3 scores (Fig 8, Table 9) with salinity is unlikely to indicate any direct effect of this variable; rather, salinity represents a marker of the hydrographical properties and history of the water masses of the different oceans and water bodies. A similar interpretation can be applied to the negative correlation between PC3 and temperature shown by the whole data set and the SCM samples (Table 9). The lack of association between PC3 and nitracline starting depths or PAR indicate that, while environmental factors are likely to have an effect, the ocean-related differences in phytoplankton community composition may respond in a large part to geographically-linked historical explanations, in agreement with the affirmation of Martiny et al. [69] that historical events leave lasting signatures on the distributions of microbial assemblages.

The highest positive loadings for PC4 (≥ 0.30) corresponded (Table 8) to nine diatom taxa, one prasinophyte (*Pterosperma moebii*) and the “Nanoflagellates (3–20 μm)”; PC4 was positively correlated with the total number of diatoms (r = 0.49, n = 406, p < 0.001) and the highest PC4 scores (Fig 6E; S2 and S3 Tables) occurred in the shallower samples of areas like the Caribbean (between stations 127–130), the Brazilian Coast (stations 25–31) and the Pacific Equatorial Upwelling region (stations 90–97). Except for this last zone, PC4 scores did not closely track Chl *a* concentrations and the correlation between this variable and the component (Table 9) was significantly negative for the whole data set. Among the taxa with relatively high (≥ 0.30) positive loadings on PC4 (hereafter the “PC4 assemblage”), the diatoms *Pseudo-nitzschia* spp., *Chaetoceros* spp. (<20 μm), *Rhizosolenia* spp. and *Planktoniella sol*, and the prasinophyte *Pterosperma moebii* had been cited by Gómez et al. [70] as typical of a group that they designed as the tropical High Nutrient Low Chlorophyll phytoplankton assemblage (HNLC-PA). Another species in the PC4 assemblage was *Hemiaulus hauckii* (with its nitrogen-fixing *Richelia intracellularis* symbiont). This species was scarce in the Pacific Equatorial Upwelling but formed a strong bloom near the Brazilian coast (S2 Fig) where it appeared to be responsible of elevated N_2_ fixation rates [43]. As found by Gómez et al. [70] for their HNLC-PA, our PC4 assemblage was more important in the shallower samples and did not include the well-silicified diatoms that typically bloom in mesotrophic coastal waters; it was also different from the community of station 45, close to the coast of South Africa (Fig 3B, 3D and 3F), in which the numerous diatoms were dominated by *Thalassiosira* spp. Gómez et al. [70] suggested that the diatoms of the HNLC-PA, which they also found in the offshore side of the Perú-Chile Current, could be better adapted to silicon deficiency and its interactions with potential iron limitation than those typical of coastal blooms. With respect to Malaspina-2010, this observation has to be interpreted in the context of the general availability of nutrients; in our case, the stations with the highest PC4 scores (Fig 6E) were associated with shallower nitraclines, higher silicate and nitrate + nitrite concentrations, and higher silicate: (nitrate+nitrite) ratios at the base of the nitracline (Kruskal-Wallis, p < 0.05) than the stations with low scores (S3 and S4 Tables). Thus, the PEQD and other zones regions in which our PC4 assemblage appeared (as happened with the HNLC-PA of [70]) could be relatively silicon-deficient when compared with some eutrophic coastal areas, but they still had higher concentrations of all major nutrients than the oligotrophic regions that were sampled during most of the Malaspina-2010 cruise. On the other hand, the association of high PC4 scores with shallower sampling depths than low scores (S2 and S3 Tables) could be related to a decrease in the efficiency of iron utilization by diatoms at sub-saturating irradiances [70,71]. PC4 was also positively correlated with temperature, both for the whole data set and for individual sampling levels (Table 9). This relationship is related to the partial association of high or low PC4 scores with certain high or low temperature provinces (such as PEQD and the Brazilian Coast) and, as found by Gómez et al. [70], should be interpreted as a consequence of the particular ecological factors of the corresponding water masses rather than a direct effect of temperature.

## Conclusions

Microscopic examination is a coarse tool to describe phytoplankton assemblages and is not adequate for picoplankton cell sizes. However, it gives insight on the phenotypic properties of a fraction of the phytoplankton community that plays a crucial role in the functioning of the planktonic food webs. In addition, because cells integrate environmental influences over periods of time ranging from days to weeks, the distribution of phytoplankton assemblages may provide valuable ecological information.

The main trends of variability discerned by the PCA highlight the contrasts between the phytoplankton assemblages of the upper and the lower euphotic zone (PC1), the composition gradients between cell-rich and cell-poor regions (PC2) and among the Atlantic, the Indian and the Pacific oceans (PC3), as well as the peculiarity of zones harbouring the diatom-dominated PC4 assemblage (PC4). These global patterns appear to reflect a combination of both environmental influences, as is mainly the case for PC1, PC2 and PC4, and historical factors, as found for PC3. In contrast, there was no sample clustering according to domains, a category that reflects the zonal variation of temperature and other climatic conditions but does not take into account geographical connections. These observations emphasize the importance of both, ecological and historical factors in shaping the distribution of phytoplankton communities.

In summary, our findings indicate that 1) assemblages of co-occurring phytoplankton taxa can be identified and 2) their distribution is best explained by a combination in different degrees of both environmental and historical influences. Obviously, the composition of phytoplankton reflects a history that is not captured in the snapshot provided by a cruise, making it difficult to find causal relationships with the measured environmental variables. However, the finding of consistent trends of variability at a global scale provides a robust framework for further ecological and biogeographical interpretation.

## Supporting Information

S1 Appendix Note on principal component analysis. (DOCX)

S1 Fig Distribution of taxa with positive or negative correlation with PC1. (A, B) taxa positively correlated with PC1. (C, D) Taxa negatively correlated with PC1 (see Table 5). (A) *Discosphaera tubifer*, (B) *Oxytoxum minutum*, (C) *Ophiaster hydroideus*, (D) *Pseudo-nitzschia* spp. For each taxon: Top, 3 m depth; centre, 20% light level; bottom, SCM depth.
(TIF)

S2 Fig Distribution of taxa with positive or negative correlation with PC2. (A, B) taxa positively correlated with PC2. (C, D) Taxa negatively correlated with PC2 (see Table 6, with the exception of *Algirosphaera robusta* and *Hemiaulus hauckii*, not included in the table because their correlation coefficients with PC2 were -0.15 and -0.13, respectively). (A) *Torodinium robustum*, (B) *Oxytoxum variabile*, (C) *Algirosphaera robusta*, (D) *Hemiaulus hauckii*. For each taxon: For each taxon: Top, 3 m depth; centre, 20% light level; bottom, SCM depth.
(TIF)

S3 Fig Distribution of phytoplankton taxa with positive or negative correlation with PC3. (A, B) taxa positively correlated with PC3. (C, D) Taxa negatively correlated with PC3 (see Table 7, with the exception of *Planktoniella sol*, not included in the table because its correlation coefficient with PC3 was -0.28). (A) *Calcidiscus leptoporus*, (B) *Helicosphaera carteri*, (C) Colonial flagellate sp. 1, (D) *Planktoniella sol*. For each taxon: Top, 3 m depth; centre, 20% light level; bottom, SCM depth.
(TIF)

S4 Fig Distribution of phytoplankton taxa with positive or negative correlation with PC4. (A, B) taxa positively correlated with PC3. (C, D) Taxa negatively correlated with PC4 (see Table 8). (A) Pennate diatom sp. 2, (B) *Pterosperma moebii*, (C) *Rhabdosphaera clavigera*, (D) *Calciopappus rigidus*. For each taxon: Top, 3 m depth; centre, 20% light level; bottom, SCM depth.
(TIF)

S5 Fig Spatial distribution of PC1 and PC2. (A, C, E) PC1. (B, D, F) PC2. Top, 3 m depth. Centre, 20% light level. Bottom, SCM depth.
(TIF)

S6 Fig Spatial distribution of PC3 and PC4. (A, C, E) PC3. (B, D, F) PC4. Top, 3 m depth. Centre, 20% light level. Bottom, SCM depth.
(TIF)

S7 Fig Relationship between PC2 and the second axis (NMDS2) of the NMDS with Chl *a* concentration for the three sampling depths. Left, relationship between PC2 and Chl *a* concentration. Right, relationship between NMDS2 and Chl a concentration. The three sampling depths (3 m, 20% light level and SCM) are indicated by different colours. The corresponding regression lines (dashed) and equations are indicated.
(TIF)

S8 Fig Distribution of the sample scores of the Atlantic, Indian and Pacific oceans in the space of PC3 and PC4. (A, D, G) Atlantic Ocean. (B, E, H) Indian Ocean. (C, F, I) Pacific Ocean. (A, B, C) 3 m depth. (D, E, F) 20% light level. (G, H, I) SCM. The letters in different colours indicate the provinces (see Table 2 for interpretation).
(TIF)

S9 Fig Position of samples in NMDS space and relationships with salinity. Left: Position of the sample points in the space of axes 1 (NMDS1) and 2 (NMDS2) of the NMDS. The numbers indicate the sampling depth (1 = 3 m, 2 = 20%, 3 = SCM). Right: Relationship between salinity and the coordinates of the sample points for the third axis (NMDS3) of the NMDS.
(TIF)

S1 Table Taxa loadings. Loadings (correlation coefficients) of the 76 taxa selected for the analysis with the first four principal components.
(DOCX)

S2 Table Samples with extreme scores for the first four components. List of the 20 samples (approximately a 5% from a total of 406) with the highest or lowest scores for each component.
(DOCX)

S3 Table Properties of samples with extreme scores for the first four components. Average ± standard deviation of principal component scores and biological and environmental variables corresponding to the samples with the highest or lowest scores for each component (listed in S2 Table).
(DOCX)

S4 Table Properties of samples with extreme scores for PC4. Average ± standard deviation of major nutrient concentrations (μmol L^-1^) and the ratio silicate: (nitrate+nitrite) at 200 m depth for the low and high PC4 samples. See S2 Table for the sample list.
(DOCX)

S5 Table Date and position of the Malaspina-2010 stations. (DOCX)
