# Supplementary material for: Phytoplankton across Tropical and Subtropical Regions of the Atlantic, Indian and Pacific Oceans
Source: PLoS One. 2016 Mar 16;11(3):e0151699. doi: 10.1371/journal.pone.0151699 (PMC4794153; doi:10.1371/journal.pone.0151699)
Supplement: S1 Appendix — (DOCX) [file pone.0151699.s001.docx]

**Appendix S1**. **Note on principal component analysis.**

Principal component analysis (PCA) is an ordination technique (Légendre and Légendre, 1989) that allows summarizing, in a reducer number of dimensions, the variance of the dispersion matrix (the correlation matrix in this work) of a set of descriptors or variables (the phytoplankton taxa) measured in a number of objects (the samples). The analysis generates a new set of variables, the principal components, which are linear combinations of the original variables and present the following properties: 1) they are uncorrelated, 2) the amount of variance explained by each succesive component is the largest possible (under the constraint of orthogonality) and is measured by the corresponding eigenvalue of the dispersion matrix. The position of the original descriptor vectors in the reduced space of the components is given by its correlation or loading on the corresponding component. Note that the points in Figure 5 represent the extremes of the taxa vectors; these should have been represented by arrows starting in the origin (0,0), which were omitted for clarity. The position of each object (sample) in the space of the components is given by its scores on the corresponding components, which was calculated from the expression:

$$V\left( j \right)= \sum_{i=1}^{n} =\frac{(x_{i}-\bar{x}_{i})F_{ij}}{s_{i}E_{j}}$$

Where *n* = number of descriptors (taxa), *V*_i_ = score of principal component *j*, *x*_i_ = abundance of taxa *i* in the sample (in our work after lograrithmic transformation), $\bar{\bar{x}_{i}}$, $s_{i}$, the mean and standard deviation of *x*_i_, $F_{ij}$= loading of species *i* on component *j*, and $E_{j}$ = eigenvalue corresponding to principal component  *j*.
