# Supplementary figures and images for: Phytoplankton across Tropical and Subtropical Regions of the Atlantic, Indian and Pacific Oceans
Source: PLoS One. 2016 Mar 16;11(3):e0151699. doi: 10.1371/journal.pone.0151699 (PMC4794153; doi:10.1371/journal.pone.0151699)

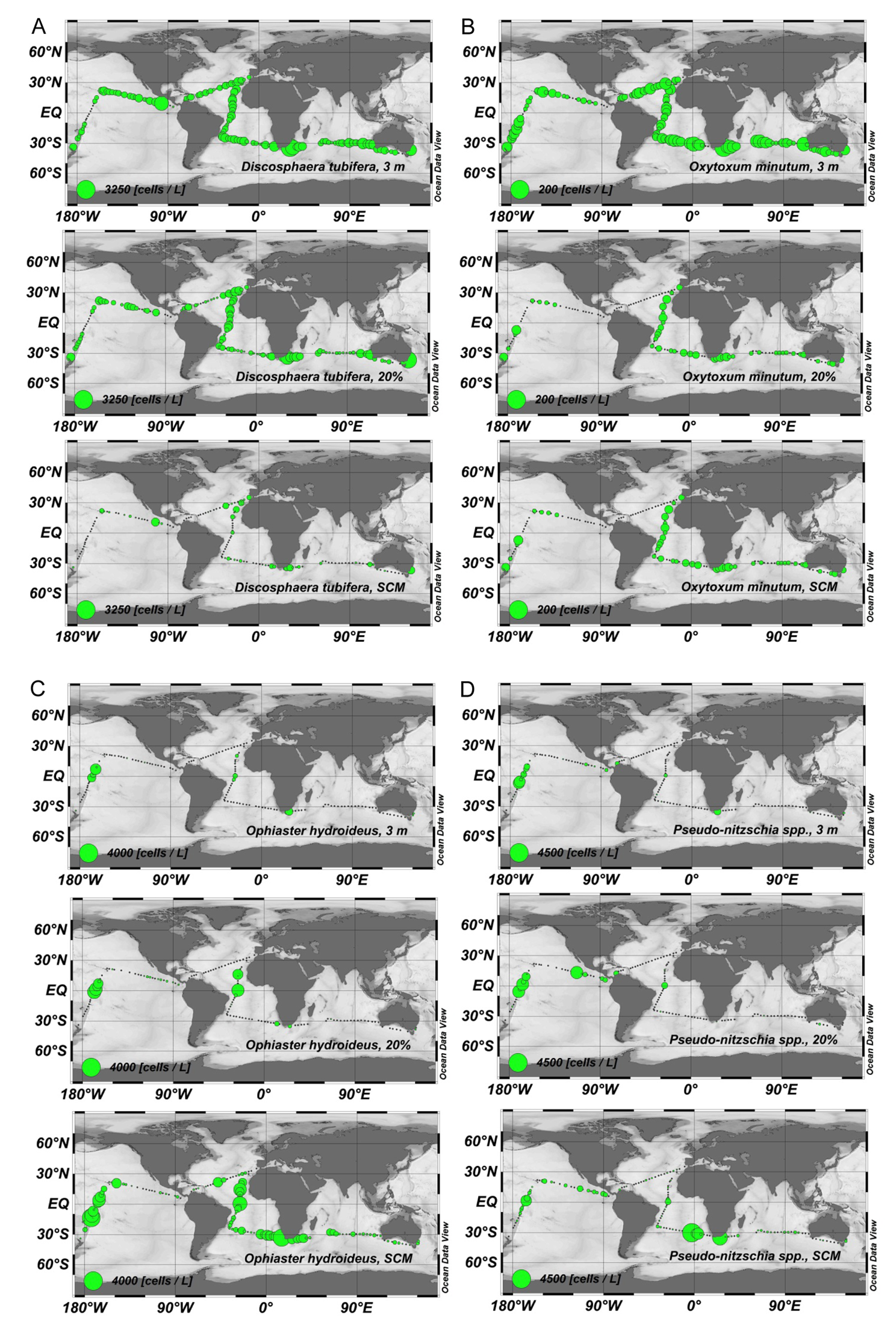

Supplement: S1 Fig — (A, B) taxa positively correlated with PC1. (C, D) Taxa negatively correlated with PC1 (see Table 5). (A) Discosphaera tubifer, (B) Oxytoxum minutum, (C) Ophiaster hydroideus, (D) Pseudo-nitzschia spp. For each taxon: Top, 3 m depth; centre, 20% light level; bottom, SCM depth. (TIF) [file pone.0151699.s002.tif]

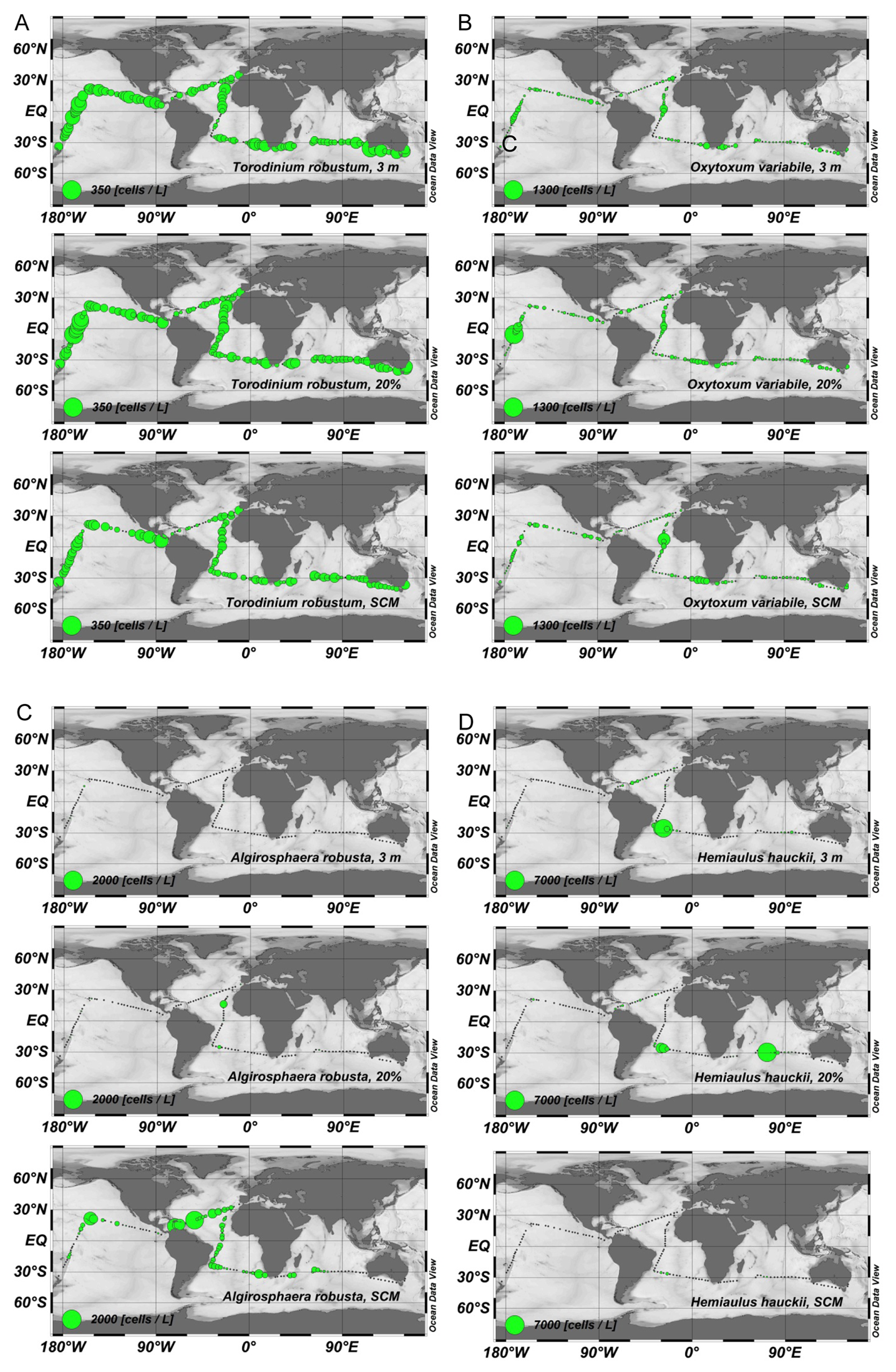

Supplement: S2 Fig — (A, B) taxa positively correlated with PC2. (C, D) Taxa negatively correlated with PC2 (see Table 6, with the exception of Algirosphaera robusta and Hemiaulus hauckii, not included in the table because their correlation coefficients with PC2 were -0.15 and -0.13, respectively). (A) Torodinium robustum, (B) Oxytoxum variabile, (C) Algirosphaera robusta, (D) Hemiaulus hauckii. For each taxon: For each taxon: Top, 3 m depth; centre, 20% light level; bottom, SCM depth. (TIF) [file pone.0151699.s003.tif]

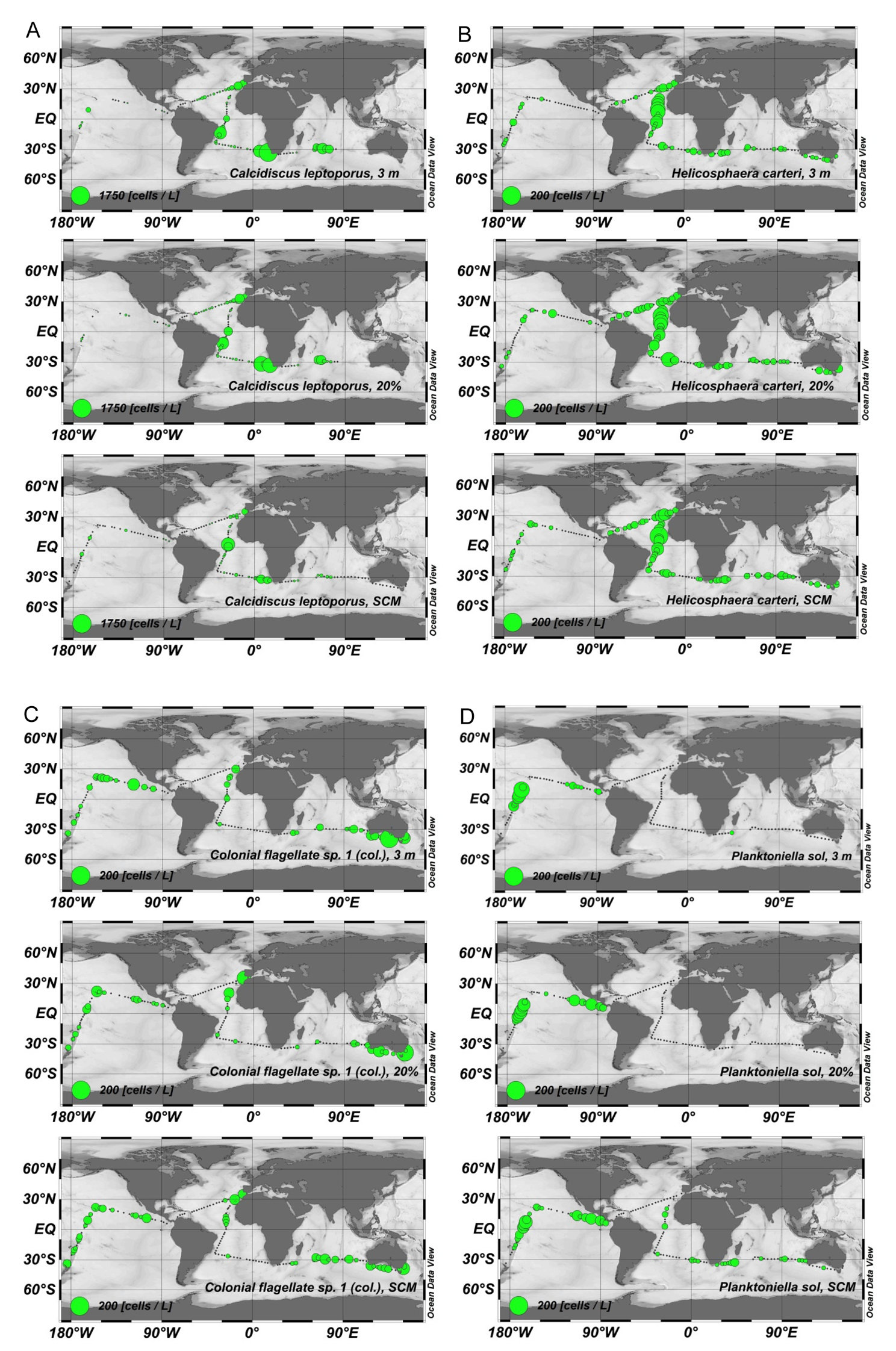

Supplement: S3 Fig — (A, B) taxa positively correlated with PC3. (C, D) Taxa negatively correlated with PC3 (see Table 7, with the exception of Planktoniella sol, not included in the table because its correlation coefficient with PC3 was -0.28). (A) Calcidiscus leptoporus, (B) Helicosphaera carteri, (C) Colonial flagellate sp. 1, (D) Planktoniella sol. For each taxon: Top, 3 m depth; centre, 20% light level; bottom, SCM depth. (TIF) [file pone.0151699.s004.tif]

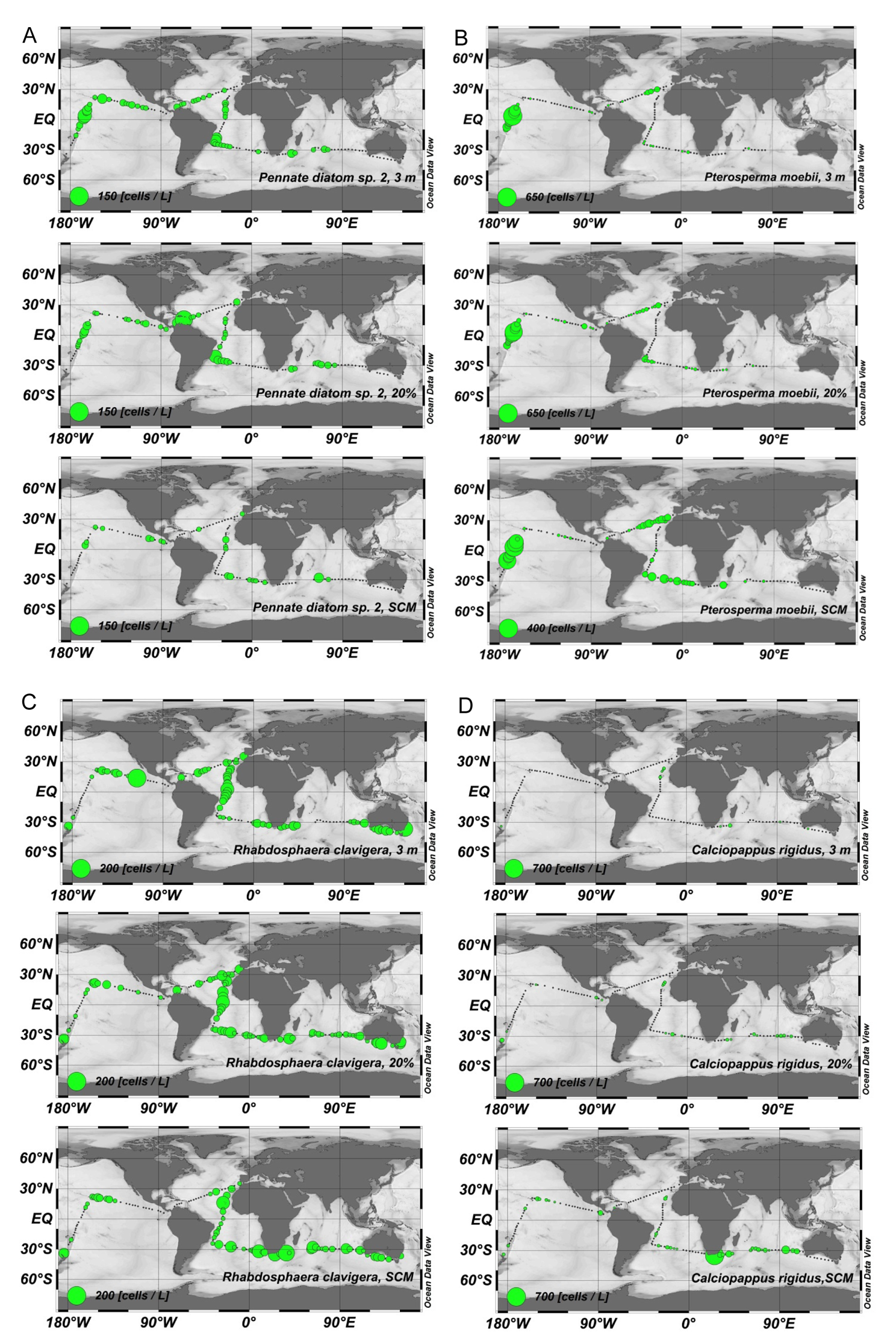

Supplement: S4 Fig — (A, B) taxa positively correlated with PC3. (C, D) Taxa negatively correlated with PC4 (see Table 8). (A) Pennate diatom sp. 2, (B) Pterosperma moebii, (C) Rhabdosphaera clavigera, (D) Calciopappus rigidus. For each taxon: Top, 3 m depth; centre, 20% light level; bottom, SCM depth. (TIF) [file pone.0151699.s005.tif]

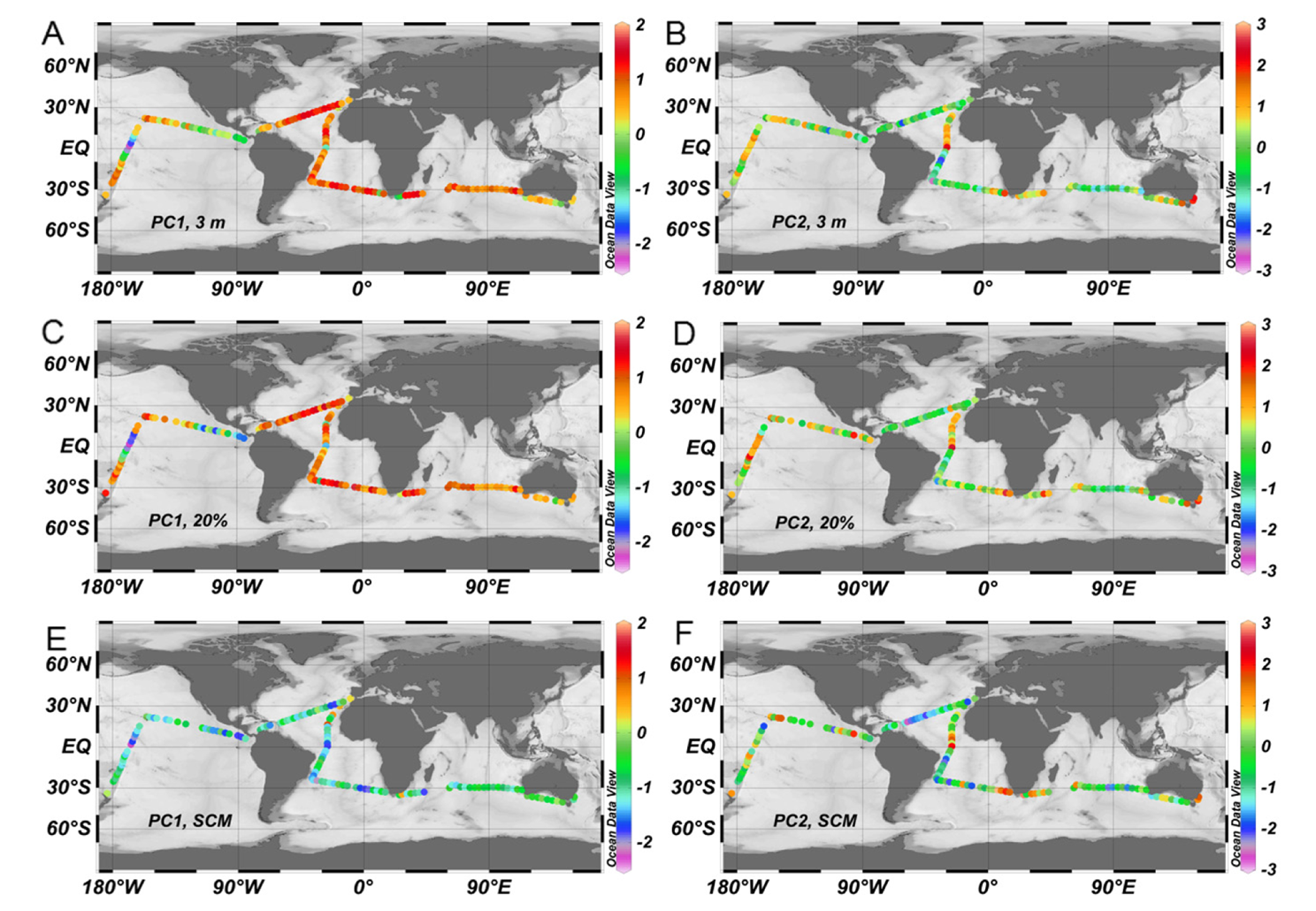

Supplement: S5 Fig — (A, C, E) PC1. (B, D, F) PC2. Top, 3 m depth. Centre, 20% light level. Bottom, SCM depth. (TIF) [file pone.0151699.s006.tif]

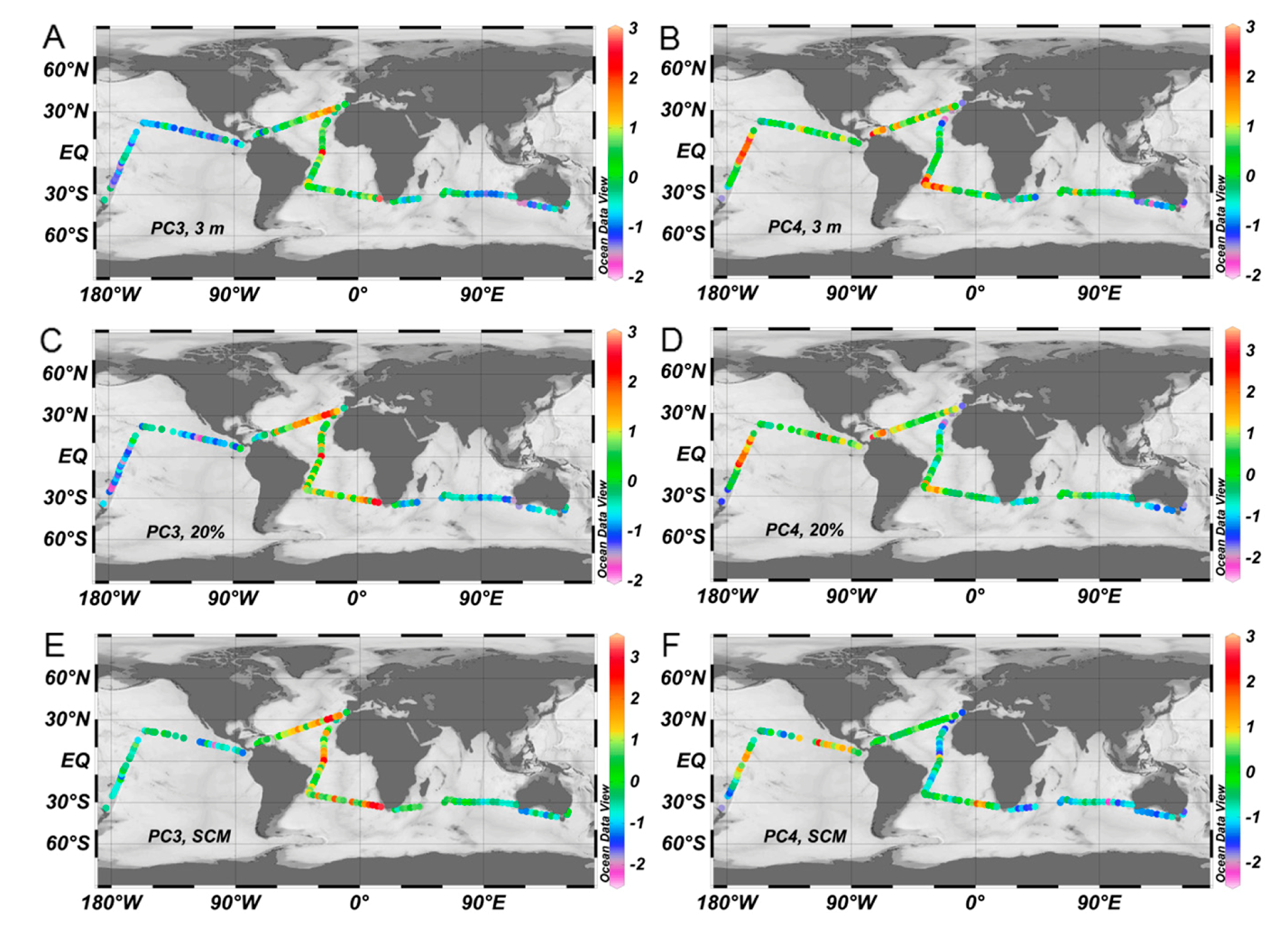

Supplement: S6 Fig — (A, C, E) PC3. (B, D, F) PC4. Top, 3 m depth. Centre, 20% light level. Bottom, SCM depth. (TIF) [file pone.0151699.s007.tif]

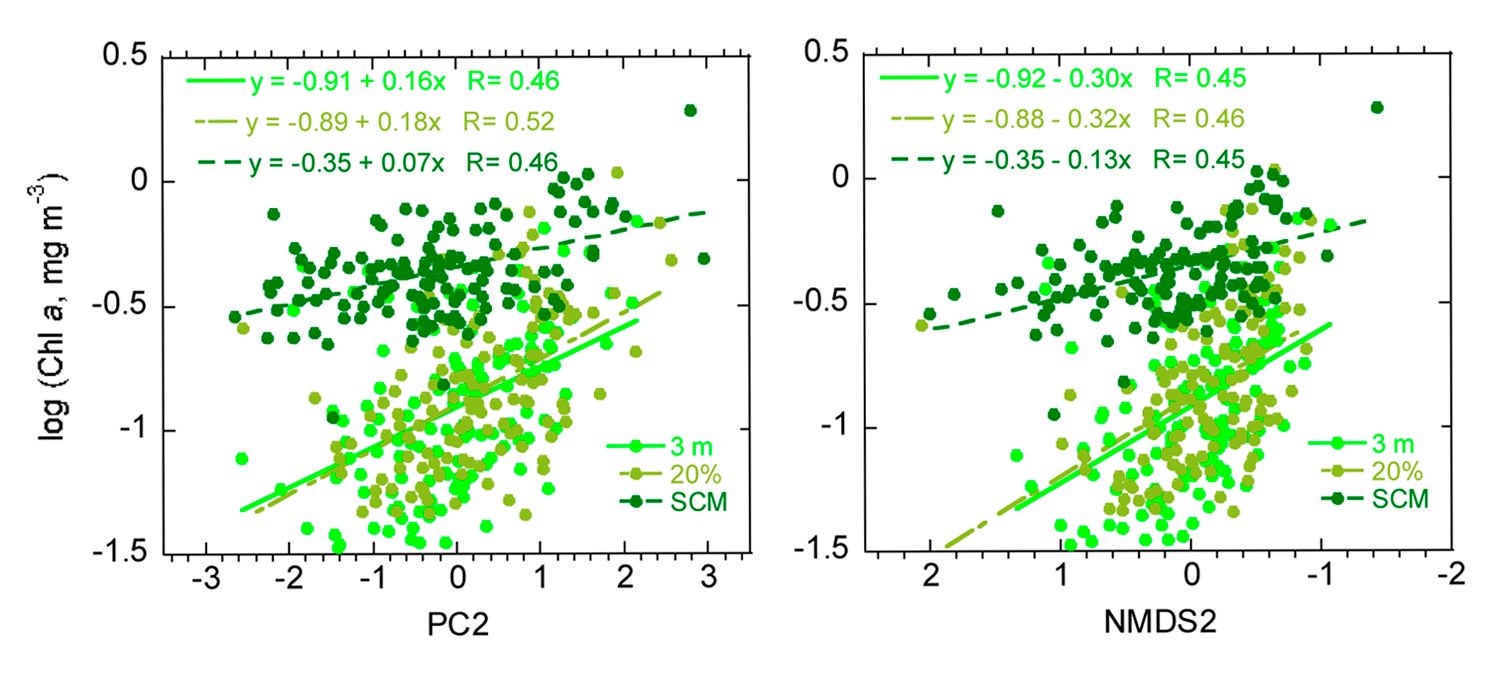

Supplement: S7 Fig — Left, relationship between PC2 and Chl a concentration. Right, relationship between NMDS2 and Chl a concentration. The three sampling depths (3 m, 20% light level and SCM) are indicated by different colours. The corresponding regression lines (dashed) and equations are indicated. (TIF) [file pone.0151699.s008.tif]

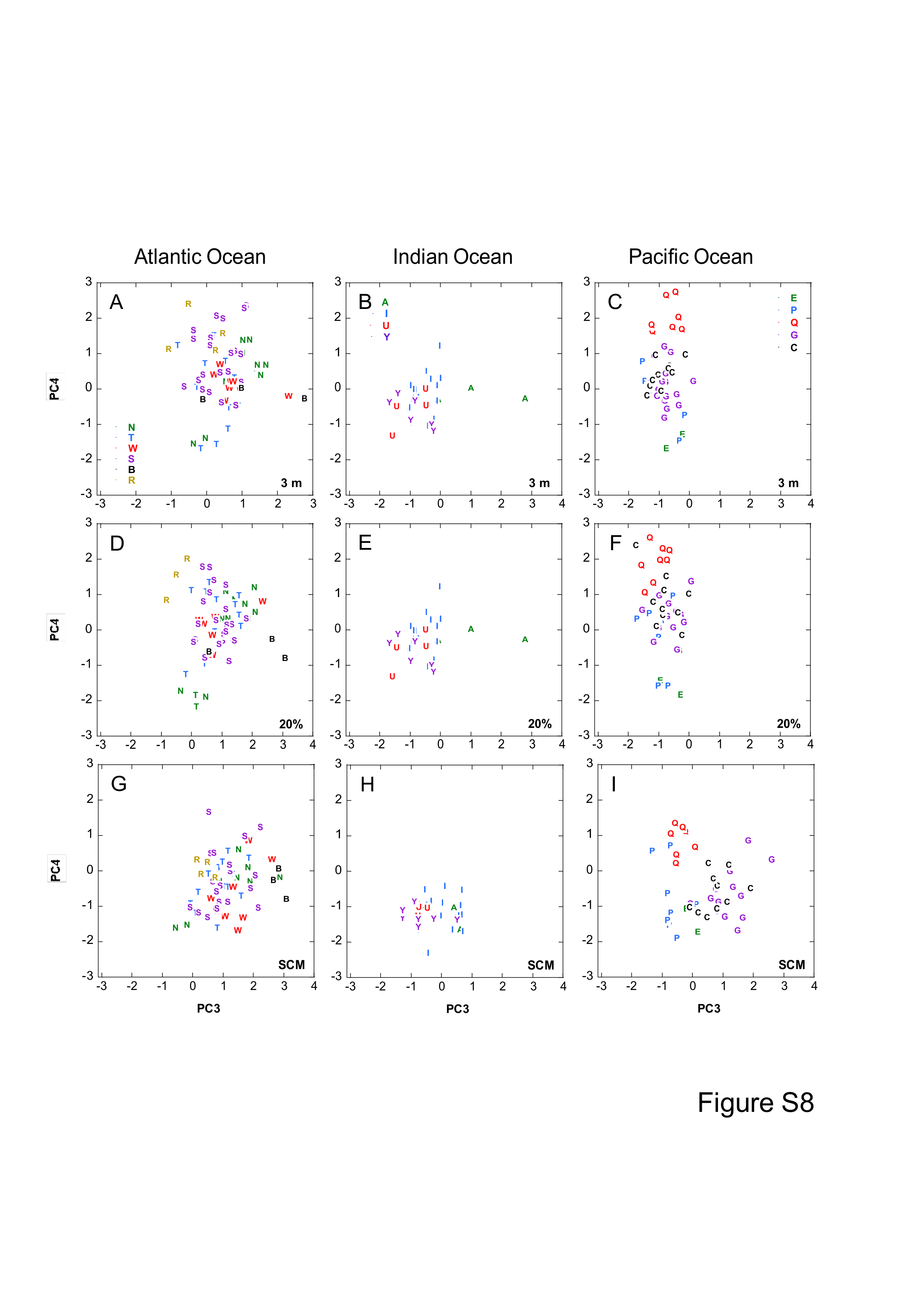

Supplement: S8 Fig — (A, D, G) Atlantic Ocean. (B, E, H) Indian Ocean. (C, F, I) Pacific Ocean. (A, B, C) 3 m depth. (D, E, F) 20% light level. (G, H, I) SCM. The letters in different colours indicate the provinces (see Table 2 for interpretation). (TIF) [file pone.0151699.s009.tif]

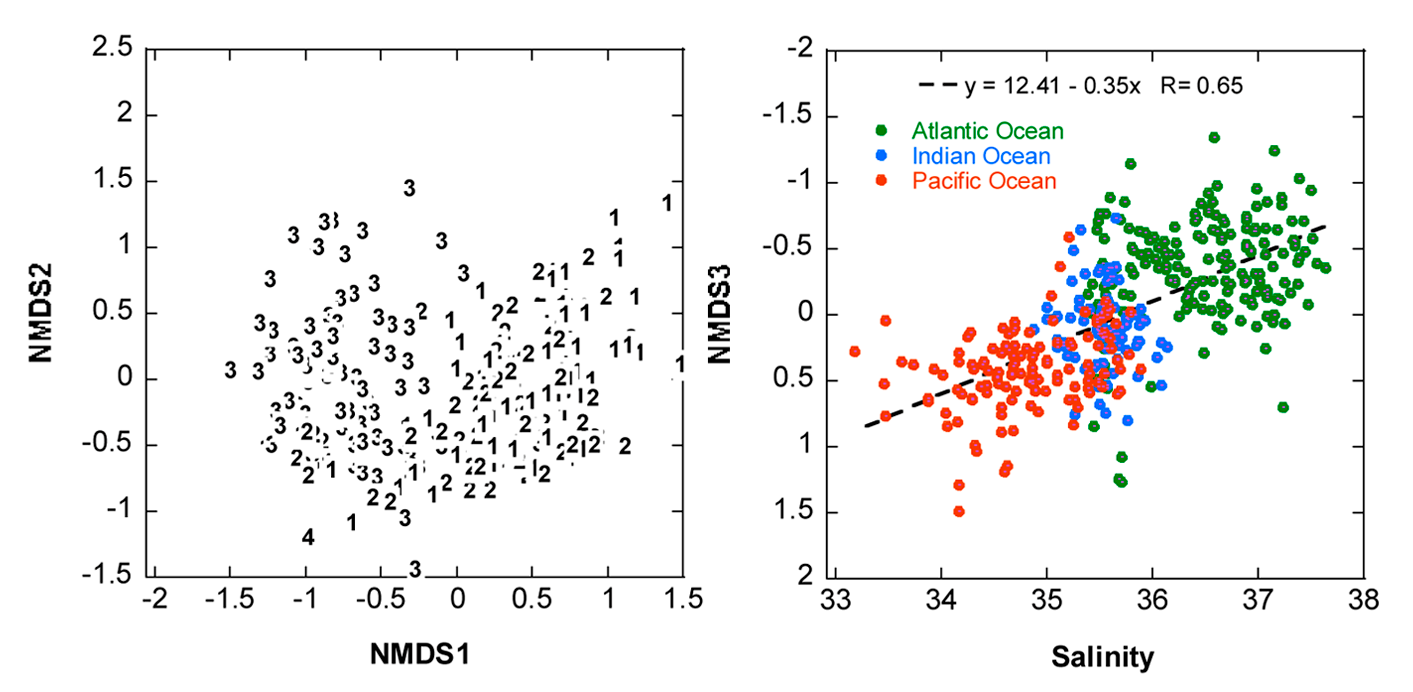

Supplement: S9 Fig — Left: Position of the sample points in the space of axes 1 (NMDS1) and 2 (NMDS2) of the NMDS. The numbers indicate the sampling depth (1 = 3 m, 2 = 20%, 3 = SCM). Right: Relationship between salinity and the coordinates of the sample points for the third axis (NMDS3) of the NMDS. (TIF) [file pone.0151699.s010.tif]
