# Supplementary material for: Phytoplankton across Tropical and Subtropical Regions of the Atlantic, Indian and Pacific Oceans
Source: PLoS One. 2016 Mar 16;11(3):e0151699. doi: 10.1371/journal.pone.0151699 (PMC4794153; doi:10.1371/journal.pone.0151699)
Supplement: S1 Table — Loadings (correlation coefficients) of the 76 taxa selected for the analysis with the first four principal components. (DOCX) [file pone.0151699.s011.docx]

**Table S1. Taxa loadings.**

| **Taxon name** | **PC1** | **PC2** | **PC3** | **PC4** |
| --- | --- | --- | --- | --- |
| DINOFLAGELLATES |  |  |  |  |
| *Gyrodinium* spp. | 0.15 | 0.63 | -0.10 | -0.10 |
| *Torodinium robustum* | -0.01 | 0.59 | -0.27 | -0.08 |
| *Gymnodinium elongatum* | 0.28 | 0.46 | 0.06 | -0.07 |
| *Oxytoxum variabile* | -0.10 | 0.56 | 0.01 | 0.02 |
| *Oxytoxum minutum* | 0.66 | 0.22 | 0.01 | -0.01 |
| *Cochlodinium* spp. | -0.01 | 0.47 | -0.08 | 0.01 |
| *Scrippsiella* spp. | 0.46 | 0.32 | 0.17 | 0.10 |
| Dinoflagellate cysts | 0.27 | 0.09 | 0.24 | 0.19 |
| *Oxytoxum* spp. | 0.27 | 0.23 | 0.16 | -0.01 |
| Unid. dinoflagellates (with inclusion bodies) | 0.09 | 0.02 | 0.64 | 0.22 |
| *Protoperidinium* spp. | 0.18 | 0.20 | 0.04 | 0.20 |
| *Pronoctiluca acuta* | 0.00 | 0.16 | -0.20 | 0.02 |
| *Ceratium teres* | 0.43 | 0.11 | 0.03 | 0.18 |
| *Oxytoxum coronatum* | 0.06 | 0.24 | 0.20 | 0.01 |
| *Gymnodinium* spp (20-40 µm) | 0.16 | 0.20 | -0.08 | 0.18 |
| *Gymnodinium* spp. (> 40 µm) | 0.06 | 0.24 | -0.29 | 0.08 |
| *Micracanthodinium claytonii* | 0.19 | 0.03 | 0.36 | 0.07 |
| *Oxytoxum scolopax* | 0.13 | 0.22 | 0.00 | -0.05 |
| *Podolampas spinifer* | 0.28 | 0.17 | 0.03 | 0.05 |
| *Oxytoxum mediterraneum* | 0.09 | 0.21 | -0.12 | 0.06 |
| *Gymnodinium* sp. ("pumpkin") | -0.35 | 0.31 | 0.18 | 0.06 |
| *Paleophalacroma unicintum* | 0.21 | 0.12 | -0.08 | -0.05 |
| *Oxytoxum longiceps* | 0.17 | 0.30 | -0.26 | -0.02 |
| *Ceratium fusus* | 0.26 | 0.17 | -0.05 | -0.15 |
| *Dinophysis* spp. (small, rounded) | 0.27 | -0.06 | 0.26 | 0.10 |
| *Gonyaulax* spp. | 0.24 | 0.15 | 0.06 | 0.05 |
| Unid. dinoflagellates (large) | 0.30 | 0.70 | -0.21 | 0.02 |
| Unid. dinoflagellates (small, < 20 µm) | 0.24 | 0.64 | 0.02 | 0.11 |
| DIATOMS |  |  |  |  |
| Unid. pennate diatoms ("benthic-like", large) | -0.13 | 0.34 | -0.24 | 0.41 |
| Unid. pennate diatoms (small, < 20 µm) | -0.09 | 0.20 | -0.06 | 0.45 |
| *Leptocylindrus mediterraneus* (with *Solenicola setigera*) | -0.17 | 0.37 | -0.10 | -0.24 |
| Unidentifed pennate diatoms | -0.74 | 0.03 | 0.10 | 0.21 |
| *Thalassiosira* spp. | -0.65 | 0.18 | 0.10 | 0.14 |
| *Pseudo-nitzschia* spp | -0.68 | 0.25 | -0.10 | 0.32 |
| *Rhizosolenia* spp. | -0.02 | 0.00 | 0.16 | 0.32 |
| Pennate diatom (sp. 2, "spindle-like") | -0.01 | 0.16 | -0.06 | 0.55 |
| Unid. centric diatoms | -0.39 | 0.09 | -0.05 | 0.15 |
| *Chaetoceros* spp. (<20 µm) | -0.43 | 0.15 | -0.22 | 0.32 |
| *Mastogloia rostrata* | 0.36 | -0.02 | -0.09 | 0.30 |
| *Hemiaulus hauckii* | 0.30 | -0.13 | 0.11 | 0.34 |
| *Planktoniella sol* | -0.57 | 0.20 | -0.28 | 0.34 |
| COCCOLITHOPHORES |  |  |  |  |
| Unid. coccolithophores (small, < 10 µm) | -0.25 | 0.58 | 0.25 | 0.11 |
| Unid. coccolithophores (large) | 0.28 | 0.26 | 0.54 | 0.01 |
| Discosphaera tubifera | 0.76 | 0.20 | 0.03 | -0.08 |
| Syracosphaera pulchra HET | 0.60 | 0.28 | 0.08 | -0.07 |
| Umbellosphaera irregularis | 0.32 | 0.33 | -0.25 | 0.09 |
| *Syracosphaera pulchra* HOL | 0.61 | 0.18 | 0.25 | 0.17 |
| *Rhabdosphaera clavigera* | 0.27 | 0.32 | 0.16 | -0.45 |
| *Helicosphaera carteri* | 0.10 | 0.09 | 0.41 | -0.30 |
| *Calciosolenia brasiliensis* | -0.56 | 0.29 | 0.19 | -0.16 |
| *Ophiaster hydroideus* | -0.76 | 0.23 | 0.14 | -0.15 |
| *Calcidiscus leptoporus* | 0.18 | 0.10 | 0.49 | 0.15 |
| *Umbilicosphaera sibogae* | -0.14 | 0.22 | 0.12 | -0.08 |
| *Calciosolenia murrayi* | -0.44 | 0.24 | 0.12 | -0.28 |
| Coccolithophore (sp. 1, "Coronosphaera-like") | 0.00 | 0.08 | 0.33 | 0.11 |
| *Acanthoica quattrospina* | 0.02 | 0.30 | -0.14 | 0.10 |
| Coccolithophore (sp. 4, "dark", 11-15 µm) | 0.39 | 0.02 | 0.38 | 0.02 |
| *Algirosphaera robusta* | -0.52 | -0.15 | 0.39 | -0.14 |
| *Umbilicosphaera anulus* | -0.39 | -0.02 | 0.34 | -0.17 |
| *Michaelsarsia elegans* | -0.40 | 0.09 | 0.35 | -0.18 |
| *Syracosphaera prolongata* | 0.05 | 0.30 | 0.03 | -0.08 |
| *Calciopappus rigidus* | -0.14 | 0.12 | 0.01 | -0.39 |
| *Oolithotus* spp. | -0.49 | 0.09 | 0.20 | 0.09 |
| Syracosphaera spp. | 0.19 | -0.02 | 0.38 | 0.07 |
| OTHER PHYTOPLANKTON |  |  |  |  |
| Cryptomonads | -0.06 | 0.17 | 0.31 | 0.13 |
| *Dictyocha fibula* | -0.08 | 0.21 | 0.39 | -0.29 |
| *Halosphaera viridis* (phycoma) | -0.31 | -0.04 | 0.36 | -0.03 |
| Colonial flagellate (sp. 1, colonies) | -0.08 | 0.25 | -0.34 | -0.37 |
| *Pterosperma* *moebii* | -0.31 | 0.07 | 0.13 | 0.45 |
| *Trichodesmium* sp. (filaments) | 0.20 | 0.09 | 0.07 | 0.07 |
| Unid. nanoflagellates (3-20 µm) | -0.09 | 0.55 | 0.29 | 0.30 |
| CILIATES |  |  |  |  |
| Ciliates ("naked", <30 µm) | -0.26 | 0.63 | 0.16 | -0.16 |
| Ciliates ( "naked", > 30 µm) | -0.11 | 0.69 | -0.30 | -0.12 |
| *Strombidium* spp. | -0.16 | 0.66 | -0.16 | -0.25 |
| Tintinnids ( large) | -0.35 | 0.35 | 0.14 | -0.02 |
| Tintinnids (< 40 µm) | -0.24 | 0.22 | 0.07 | 0.00 |

Unid. = Unidentified. Loadings (correlation coefficients) of the 76 taxa selected for the analysis with the first four principal components.
