# Supplementary material for: Phytoplankton across Tropical and Subtropical Regions of the Atlantic, Indian and Pacific Oceans
Source: PLoS One. 2016 Mar 16;11(3):e0151699. doi: 10.1371/journal.pone.0151699 (PMC4794153; doi:10.1371/journal.pone.0151699)
Supplement: S2 Table — List of the 20 samples (approximately a 5% from a total of 406) with the highest or lowest scores for each component. (DOCX) [file pone.0151699.s012.docx]

**Table S2.** **Samples with extreme scores for the first four components.**

|  | **List of samples** |
| --- | --- |
| **Sample group** | **Province (Station - number of depths)** |
| Low PC1* Except PEQD (n = 9) | WTRA (16), SATL (28, 39, 40), ISSG (51), PNEC (119, 124, 125), NASE (145) |
| Low PC1* PEQD (n = 11) | PEQD (93-2, 94-3, 95-3, 96-2, 97) |
| High PC1 | WTRA (12), SATL (32, 33-2, 37, 40, 41), ISSG (49-2, 50), AUSW (67), NATR (135, 138, 139), NASE (140-2, 141, 143-2, 146) |
| Low PC2 | SATL (20-2, 21-2, 27-2, 30, 35), ISSG (60), NPTG (99, 113, 114), PNC (117-3), NATR (13-2, 133, 137), NASE (147) |
| High PC2 | WTRA (14, 16-3), BENG (43-2, 44), EAFR (45), ISSG (50), SSTC (76), AUSE (77-2, 78-2), PEQD (90, 91), NPTG (104), PNEC (122-2) |
| Low PC3 | ISSG (62), AUSW (66, 68, 69-2), SSTC (70), SPSG (83-2, 84, 85, 88, 89), PEQD (92, 96), NPTG (114, 115), PNEC (116-2, 119, 121) |
| High PC3 | WTRA (16-3), SATL (33, 37, 41), BENG (42, 43-2, 44-3), NATR (137), NASE (140, 142, 144-2, 145-2) |
| Low PC4 | NASE (1, 3-2, 6-3), NATR (7), SATL (14), EAFR (46, 47), ISSG (49, 50, 63, 65), SSTC (72, 73), AUSE (77, 78-2), SPSG (79-2) |
| High PC4* except PEQD (n = 10) | SATL (25-2, 26, 29, 31), PNEC (116-2), CARB (127-2, 130) |
| High PC4 * PEQD (n = 10) | PEQD (90, 91-2, 92, 93-2, 94, 96, 97-2) |

List of the 20 samples (approximately a 5% from a total of 406) with the highest or lowest scores for each component. *In the case of PC1 low and PC4 high, the calculations were carried out separately for the samples from the PEQD (Pacific equatorial Upwelling Province) and the other provinces.
