# Supplementary material for: Phytoplankton across Tropical and Subtropical Regions of the Atlantic, Indian and Pacific Oceans
Source: PLoS One. 2016 Mar 16;11(3):e0151699. doi: 10.1371/journal.pone.0151699 (PMC4794153; doi:10.1371/journal.pone.0151699)
Supplement: S3 Table — Average ± standard deviation of principal component scores and biological and environmental variables corresponding to the samples with the highest or lowest scores for each component (listed in S2 Table). (DOCX) [file pone.0151699.s013.docx]

**Table S3**. **Properties of samples with extreme scores on the first four components.**

| **Sample group** | **PC Score** | **Depth code** | **Depth** | **Dinoflage-llates** | **Diatoms** | **Coccolitho-phores** | **Chl *a*** | **MLD depth** | **Nitracline depth** | **Z_1%_** |
| --- | --- | --- | --- | --- | --- | --- | --- | --- | --- | --- |
|  |  |  | **m** | **cells L^-1^** | **cells L^-1^** | **cells L^-1^** | **mg m^-3^** | **m** | **m** | **m** |
| Low PC1 Except PEQD | -1.87 ± 0.09 | 2.9 ± 0.3 | 70.6 ± 40.5 | 2169 ± 1183 | 1607 ± 1039 | 10036 ± 9902 | 0.55 ± 0.17 | 40 ± 23 | 29 ± 28 | 86 ± 20 |
| Low PC1 PEQD | -2 ± 0.19 | 2 ± 0.8 | 37.0 ± 30.5 | 2325 ± 963 | 1729 ± 896 | 24658 ± 7355 | 0.32 ± 0.05 | 92 ± 14 | 16 ± 23 | 90 ± 4 |
| High PC1 | 1.4 ± 0.08 | 1.4 ± 0.5 | 17.8 ± 19.5 | 3937 ± 1431 | 226 ± 201 | 6513 ± 2914 | 0.08 ± 0.04 | 40 ± 10 | 82 ± 50 | 109 ± 26 |
| Low PC2 | -2.16 ± 0.32 | 2.5 ± 0.9 | 94.6 ± 63.4 | 797 ± 407 | 234 ± 187 | 3778 ± 2511 | 0.33 ± 0.17 | 47 ± 19 | 119 ± 43 | 125 ± 35 |
| High PC2 | 2.02 ± 0.39 | 2.2 ± 0.8 | 38.8 ± 31.2 | 6371 ± 2915 | 1628 ± 2099 | 24282 ± 12422 | 0.60 ± 0.41 | 33 ± 20 | 48 ± 48 | 87 ± 23 |
| Low PC3 | -1.56 ± 0.18 | 1.7 ± 0.7 | 29.6 ± 34.7 | 2983 ± 1199 | 992 ± 1385 | 5072 ± 3797 | 0.27 ± 0.23 | 49 ± 19 | 63 ± 31 | 100 ± 18 |
| High PC3 | 2.35 ± 0.38 | 2.5 ± 0.7 | 68.1 ± 45.0 | 4270 ± 3054 | 678 ± 938 | 18816 ± 13200 | 0.46 ± 0.27 | 43 ± 15 | 58 ± 55 | 105 ± 31 |
| Low PC4 | -1.75 ± 0.19 | 2.3 ± 0.8 | 56.1 ± 41.5 | 2966 ± 1329 | 270 ± 209 | 11750 ± 11082 | 0.35 ± 0.22 | 59 ± 20 | 72 ± 27 | 97 ± 22 |
| High PC4 Except PEQD | 2.25 ± 0.36 | 1.6 ± 0.7 | 20.7 ± 22.8 | 2819 ± 1597 | 4061 ± 3540 | 5611 ± 2389 | 0.20 ± 0.20 | 43 ± 8 | 50 ± 49 | 103 ± 33 |
| High PC4 PEQD | 2.22 ± 0.35 | 1.6 ± 0.5 | 22.1 ± 16.8 | 5422 ± 3019 | 2068 ± 870 | 20938 ± 8282 | 0.29 ± 0.20 | 79 ± 23 | 12 ± 21 | 97 ± 12 |

Average ± standard deviation of principal component scores and biological and environmental variables corresponding to the samples with the highest or lowest scores for each component (listed in Table S2). Depth codes indicate the nominal sampling depth; its values are 1 for surface, 2 for the 20% light level and 3 for the SCM. Nitracline depth is the strating depth of the nitraclina and Z_1%_ the depth of the 1% light level.
