# Supplementary material for: Phytoplankton across Tropical and Subtropical Regions of the Atlantic, Indian and Pacific Oceans
Source: PLoS One. 2016 Mar 16;11(3):e0151699. doi: 10.1371/journal.pone.0151699 (PMC4794153; doi:10.1371/journal.pone.0151699)
Supplement: S4 Table — Average ± standard deviation of major nutrient concentrations (μmol L-1) and the ratio silicate: (nitrate+nitrite) at 200 m depth for the low and high PC4 samples. See S2 Table for the sample list. (DOCX) [file pone.0151699.s014.docx]

**Table S4**. **Properties of samples with extreme scores for PC4*.**

| **Sample group** | **Silicate** | **Nitrate+nitrite** | **Phosphate** | **Silicate: (Nitrate+nitrite)** |
| --- | --- | --- | --- | --- |
| Low PC4 | 3.59 ± 2.51 | 8.69 ± 3.74 | 0.68 ± 0.40 | 0.40 ± 0.20 |
| High PC4 Except PEQD | 8.76 ± 12.97 | 10.17 ± 9.10 | 0.82 ± 1.02 | 0.91 ± 0.60 |
| High PC4 PEQD | 19.63 ± 11.61 | 18.97 ± 6.96 | 1.18 ± 0.49 | 0.96 ± 0.33 |

Average ± standard deviation of major nutrient concentrations (µmol L^-1^) and the ratio silicate: (nitrate+nitrite) at 200 m depth for the low and high PC4 samples. * See Table S2 for the sample list.
