# Supplementary material for: Phytoplankton across Tropical and Subtropical Regions of the Atlantic, Indian and Pacific Oceans
Source: PLoS One. 2016 Mar 16;11(3):e0151699. doi: 10.1371/journal.pone.0151699 (PMC4794153; doi:10.1371/journal.pone.0151699)
Supplement: S5 Table — (DOCX) [file pone.0151699.s015.docx]

**S5. Table. Date and position of the Malaspina-2010 stations.**

| Date | Station number | Latitude | Longitude |
| --- | --- | --- | --- |
| 16-12-2010 | 1 | 35 08.12 N | 09 30.99 W |
| 17-12-2010 | 2 | 34 00.04 N | 12 46.83 W |
| 19-12-2010 | 3 | 29 41.00 N | 17 17.28 W |
| 20-12-2010 | 4 | 28 42.10 N | 18 52.27 W |
| 21-12-2010 | 5 | 24 58.30 N | 21 03.19 W |
| 22-12-2010 | 6 | 23 16.44 N | 22 13.33 W |
| 23-12-2010 | 7 | 21 27.01 N | 23 27.27 W |
| 24-12-2010 | 8 | 20 15.67 N | 24 15.07 W |
| 25-12-2010 | 9 | 16 09.84 N | 26 01.53 W |
| 26-12-2010 | 10 | 14 31.18 N | 26 00.02 W |
| 27-12-2010 | 11 | 12 29.90 N | 25 59.17 W |
| 28-12-2010 | 12 | 09 33.82 N | 25 59.60 W |
| 29-12-2010 | 13 | 07 19.41 N | 25 59.75 W |
| 30-12-2010 | 14 | 04 59.32 N | 26 00.29 W |
| 31-12-2010 | 15 | 02 27.44 N | 26 00.54 W |
| 01-01-2011 | 16 | 00 10.86 N | 26 00.91 W |
| 02-01-2011 | 17 | 03 01.77 S | 27 19.54 W |
| 03-01-2011 | 18 | 04 46.72 S | 28 10.12 W |
| 04-01-2011 | 19 | 07 16.99 S | 29 19.34 W |
| 05-01-2011 | 20 | 09 07.65 S | 30 11.29 W |
| 06-01-2011 | 21 | 11 38.43 S | 31 22.32 W |
| 07-01-2011 | 22 | 13 44.13 S | 32 22.57 W |
| 08-01-2011 | 23 | 15 49.85 S | 33 24.19 W |
| 09-01-2011 | 24 | 18 25.45 S | 34 38.66 W |
| 10-01-2011 | 25 | 21 05.60 S | 35 58.34 W |
| 11-01-2011 | 26 | 22 57.28 S | 36 55.48 W |
| 19-01-2011 | 27 | 24 18.90 S | 36 13.33 W |
| 20-01-2011 | 28 | 24 47.52 S | 33 28.56 W |
| 21-01-2011 | 29 | 25 25.59 S | 30 04.19 W |
| 22-01-2011 | 30 | 25 52.09 S | 27 34.04 W |
| 23-01-2011 | 31 | 26 27.92 S | 24 12.58 W |
| 24-01-2011 | 32 | 26 56.81 S | 21 23.99 W |
| 25-01-2011 | 33 | 27 33.20 S | 18 05.36 W |
| 26-01-2011 | 34 | 28 05.44 S | 14 47.48 W |
| 27-01-2011 | 35 | 28 38.94 S | 11 48.70 W |
| 28-01-2011 | 36 | 29 06.34 S | 09 07.85 W |
| 29-01-2011 | 37 | 29 46.04 S | 05 18.89 W |
| 30-01-2011 | 38 | 30 15.70 S | 02 26.12 W |
| 31-01-2011 | 39 | 30 52.90 S | 00 58.07 E |
| 01-02-2011 | 40 | 31 18.45 S | 03 44.92 E |
| 02-02-2011 | 41 | 31 49.94 S | 06 51.42 E |
| 03-02-2011 | 42 | 32 14.05 S | 09 20.71 E |
| 04-02-2011 | 43 | 32 48.76 S | 12 46.15 E |
| 05-02-2011 | 44 | 33 14.00 S | 15 20.37 E |
| 13-02-2011 | 45 | 35 08.17 S | 25 33.79 E |
| 14-02-2011 | 46 | 34 50.24 S | 27 32.95 E |
| 15-02-2011 | 47 | 34 26.38 S | 31 06.71 E |
| 16-02-2011 | 48 | 34 10.43 S | 33 43.55 E |
| 17-02-2011 | 49 | 33 54.43 S | 37 02.53 E |
| 18-02-2011 | 50 | 33 33.75 S | 39 53.07 E |
| 19-02-2011 | 51 | 33 11.66 S | 43 14.89 E |
| 24-02-2011 | 52 | 30 03.30 S | 61 25.84 E |
| 25-02-2011 | 53 | 27 58.69 S | 63 14.86 E |
| 26-02-2011 | 54 | 28 07.65 S | 66 29.59 E |
| 27-02-2011 | 55 | 29 21.21 S | 69 24.00 E |
| 28-02-2011 | 56 | 29 33.63 S | 72 26.65 E |
| 01-03-2011 | 57 | 29 54.26 S | 76 05.60 E |
| 02-03-2011 | 58 | 29 49.65 S | 79 36.66 E |
| 03-03-2011 | 59 | 29 48.63 S | 82 37.40 E |
| 04-03-2011 | 60 | 29 44.93 S | 86 15.39 E |
| 05-03-2011 | 61 | 29 41.68 S | 89 29.91 E |
| 06-03-2011 | 62 | 29 37.61 S | 92 59.05 E |
| 07-03-2011 | 63 | 29 34.44 S | 96 24.44 E |
| 08-03-2011 | 64 | 30 19.96 S | 103 18.45 E |
| 09-03-2011 | 65 | 30 19.65 S | 103 19.04 E |
| 10-03-2011 | 66 | 30 48.63 S | 107 14.23 E |
| 11-03-2011 | 67 | 31 08.04 S | 110 12.42 E |
| 12-03-2011 | 68 | 31 33.22 S | 113 28.01 E |
| 19-03-2011 | 69 | 35 59.00 S | 117 24.42 E |
| 20-03-2011 | 70 | 36 38.36 S | 120 51.37 E |
| 21-03-2011 | 71 | 37 14.36 S | 124 53.87 E |
| 22-03-2011 | 72 | 37 53.36 S | 127 45.02 E |
| 23-03-2011 | 73 | 38 34.65 S | 131 30.39 E |
| 24-03-2011 | 74 | 39 14.55 S | 135 08.33 E |
| 25-03-2011 | 75 | 39 52.01 S | 138 44.12 E |
| 26-03-2011 | 76 | 40 33.02 S | 142 29.82 E |
| 27-03-2011 | 77 | 38 37.28 S | 150 25.04 E |
| 28-03-2011 | 78 | 36 38.50 S | 151 00.37 E |
| 16-04-2011 | 79 | 34 03.37 S | 176 00.95 E |
| 17-04-2011 | 80 | 34 03.29 S | 176 00.34 E |
| 18-04-2011 | 81 | 28 24.37 S | 179 08.48 E |
| 19-04-2011 | 82 | 25 29.39 S | 179 31.42 W |
| 20-04-2011 | 83 | 23 22.63 S | 178 12.65 W |
| 21-04-2011 | 84 | 20 39.66 S | 176 55.18 W |
| 22-04-2011 | 85 | 18 33.65 S | 175 49.40 W |
| 23-04-2011 | 86 | 15 54.18 S | 174 29.21 W |
| 24-04-2011 | 87 | 13 31.87 S | 173 22.31 W |
| 25-04-2011 | 88 | 11 14.52 S | 172 37.78 W |
| 26-04-2011 | 89 | 09 28.45 S | 172 17.88 W |
| 27-04-2011 | 90 | 07 03.63 S | 171 23.43 W |
| 28-04-2011 | 91 | 05 45.00 S | 170 44.44 W |
| 29-04-2011 | 92 | 03 24.62 S | 169 27.75 W |
| 30-04-2011 | 93 | 01 18.19 S | 168 21.24 W |
| 01-05-2011 | 94 | 01 35.93 N | 166 50.97 W |
| 02-05-2011 | 95 | 03 44.89 N | 165 46.39 W |
| 03-05-2011 | 96 | 06 59.53 N | 164 22.30 W |
| 04-05-2011 | 97 | 09 12.07 N | 163 29.70 W |
| 05-05-2011 | 98 | 11 35.55 N | 162 24.57 W |
| 06-05-2011 | 99 | 14 59.81 N | 160 48.96 W |
| 07-05-2011 | 100 | 17 58.35 N | 159 26.07 W |
| 14-05-2011 | 101 | 21 53.40 N | 155 39.83 W |
| 15-05-2011 | 102 | 21 34.20 N | 153 25.09 W |
| 16-05-2011 | 103 | 21 03.83 N | 150 19.14 W |
| 17-05-2011 | 104 | 20 47.01 N | 148 16.15 W |
| 18-05-2011 | 105 | 20 20.67 N | 145 11.83 W |
| 19-05-2011 | 106 | 19 53.49 N | 141 35.40 W |
| 20-05-2011 | 107 | 19 16.74 N | 138 57.91 W |
| 21-05-2011 | 108 | 18 39.19 N | 136 10.69 W |
| 22-05-2011 | 109 | 18 01.67 N | 133 15.59 W |
| 23-05-2011 | 110 | 17 22.22 N | 130 36.66 W |
| 24-05-2011 | 111 | 16 37.50 N | 127 32.17 W |
| 25-05-2011 | 112 | 15 54.52 N | 124 28.43 W |
| 26-05-2011 | 113 | 15 18.62 N | 121 59.72 W |
| 27-05-2011 | 114 | 14 31.71 N | 118 46.42 W |
| 28-05-2011 | 115 | 13 48.23 N | 115 45.37 W |
| 29-05-2011 | 116 | 13 12.30 N | 113 15.36 W |
| 30-05-2011 | 117 | 12 29.71 N | 110 23.74 W |
| 31-05-2011 | 118 | 12 00.04 N | 108 04.56 W |
| 01-06-2011 | 119 | 11 21.26 N | 105 00.64 W |
| 02-06-2011 | 120 | 10 45.51 N | 102 26.54 W |
| 03-06-2011 | 121 | 10 05.55 N | 99 14.77 W |
| 04-06-2011 | 122 | 09 26.74 N | 96 20.44 W |
| 05-06-2011 | 123 | 08 44.74 N | 93 08.66 W |
| 06-06-2011 | 124 | 08 08.51 N | 90 21.90 W |
| 07-06-2011 | 125 | 07 13.42 N | 87 57.59 W |
| 08-06-2011 | 126 | 05 54.69 N | 84 47.68 W |
| 20-06-2011 | 127 | 12 21.76 N | 74 02.35 W |
| 21-06-2011 | 128 | 14 09.48 N | 71 40.40 W |
| 22-06-2011 | 129 | 15 04.11 N | 69 17.72 W |
| 23-06-2011 | 130 | 15 31.50 N | 67 00.86 W |
| 25-06-2011 | 131 | 17 25.63 N | 59 49.66 W |
| 26-06-2011 | 132 | 18 03.84 N | 57 48.20 W |
| 27-06-2011 | 133 | 18 59.77 N | 55 09.01 W |
| 28-06-2011 | 134 | 19 59.38 N | 52 38.19 W |
| 29-06-2011 | 135 | 20 48.23 N | 50 08.59 W |
| 30-06-2011 | 136 | 21 44.13 N | 47 47.26 W |
| 01-07-2011 | 137 | 22 51.75 N | 44 31.68 W |
| 02-07-2011 | 138 | 23 44.11 N | 41 54.11 W |
| 03-07-2011 | 139 | 24 50.67 N | 38 42.61 W |
| 04-07-2011 | 140 | 26 06.61 N | 35 15.45 W |
| 05-07-2011 | 141 | 26 54.66 N | 32 50.19 W |
| 06-07-2011 | 142 | 27 58.01 N | 29 39.13 W |
| 07-07-2011 | 143 | 28 52.47 N | 26 57.04 W |
| 08-07-2011 | 144 | 29 58.01 N | 23 41.14 W |
| 09-07-2011 | 145 | 30 57.96 N | 20 38.99 W |
| 10-07-2011 | 146 | 32 04.73 N | 17 15.96 W |
| 11-07-2011 | 147 | 32 54.86 N | 14 36.70 W |
